# DSCT: a novel deep-learning framework for rapid and accurate spatial transcriptomic cell typing

**DOI:** 10.1093/nsr/nwaf030

**Published:** 2025-01-28

**Authors:** Yiheng Xu, Bin Yu, Xuan Chen, Aibing Peng, Quyuan Tao, Youzhe He, Yueming Wang, Xiao-Ming Li

**Affiliations:** Department of Neurology and Department of Psychiatry, the Second Affiliated Hospital, Zhejiang University School of Medicine, Hangzhou 310058, China; NHC and CAMS Key Laboratory of Medical Neurobiology, MOE Frontier Center of Brain Science and Brain-machine Integration, School of Brain Science and Brain Medicine, Zhejiang University, Hangzhou 310058, China; College of Computer Science and Technology, Zhejiang University, Hangzhou 310000, China; Key Laboratory of Novel Targets and Drug Study for Neural Repair of Zhejiang Province, Institute of brain and cognitive science, Hangzhou City University School of Medicine, Hangzhou 310015, China; NHC and CAMS Key Laboratory of Medical Neurobiology, MOE Frontier Center of Brain Science and Brain-machine Integration, School of Brain Science and Brain Medicine, Zhejiang University, Hangzhou 310058, China; Department of Neurobiology, Affiliated Mental Health Center & Hangzhou Seventh People's Hospital, Zhejiang University School of Medicine, Hangzhou 310058, China; NHC and CAMS Key Laboratory of Medical Neurobiology, MOE Frontier Center of Brain Science and Brain-machine Integration, School of Brain Science and Brain Medicine, Zhejiang University, Hangzhou 310058, China; BGI Research, Hangzhou 310030, China; BGI Research, Hangzhou 310030, China; College of Computer Science and Technology, Zhejiang University, Hangzhou 310000, China; Qiushi Academy for Advanced Studies, Zhejiang University, Hangzhou 310058, China; The Nanhu Brain-computer Interface institute, Hangzhou 311100, China; Department of Neurology and Department of Psychiatry, the Second Affiliated Hospital, Zhejiang University School of Medicine, Hangzhou 310058, China; NHC and CAMS Key Laboratory of Medical Neurobiology, MOE Frontier Center of Brain Science and Brain-machine Integration, School of Brain Science and Brain Medicine, Zhejiang University, Hangzhou 310058, China

**Keywords:** DSCT, spatial cell typing, spatial transcriptomics, deep-learning neural network

## Abstract

Unraveling complex cell-type-composition and gene-expression patterns at the cellular spatial resolution is crucial for understanding intricate cell functions in the brain. In this study, we developed Deep Neural Network-based Spatial Cell Typing (DSCT)—an innovative framework for spatial cell typing within spatial transcriptomic data sets. This approach utilizes a synergistic integration of an enhanced gene-selection strategy and a lightweight deep neural network for data training, offering a more rapid and accurate solution for the analysis of spatial transcriptomic data. Based on comprehensive analysis, DSCT achieved exceptional accuracy in cell-type identification across various brain regions, species and spatial transcriptomic platforms. It also performed well in mapping finer cell types, thereby showcasing its versatility and adaptability across diverse data sets. Strikingly, DSCT exhibited high efficiency and remarkable processing speed, with fewer computational resource demands. As such, this novel approach opens new avenues for exploring the spatial organization of cell types and gene-expression patterns, advancing our understanding of biological functions and pathologies within the nervous system.

## INTRODUCTION

Cellular composition, spatial organization and gene-expression profiles within complex biological tissues are fundamental to understanding their biological function and pathological states. Recent advancements in spatial transcriptomic technologies have unlocked the potential to map genome-wide gene-expression patterns across tissues at single-cell and even subcellular resolutions [1–4]. Despite their transformative potential, existing technologies each addresses only certain aspects of the underlying challenges, with inherent limitations in scope and resolution. *In situ* hybridization technologies, such as MERFISH [5], smFISH (osmFISH) [6] and sequential FISH (seqFISH+) [7], offer high resolution but are confined to detecting a predefined set of genes. Similarly, *in situ* sequencing approaches, such as FISSEQ [8], BaristaSeq [9] and STARmap [10], require predefined gene panels that are specific to tissue types, limiting their adaptability. In contrast, *in situ* capturing technologies, such as spatial transcriptomics ST/Visium [11], Slide-seq [12] and Stereo-seq [13], offer near-comprehensive transcriptome coverage but generally exhibit low capture efficiency and spatial resolutions at or above the single-cell scale. To address these limitations, recent strategies have centered on computationally integrating spatial measurements with single-cell transcriptomic data [14,15]. These techniques leverage cell-type signatures or variable genes that are identified through single-cell ribonucleic acid (RNA) sequencing (scRNA-seq), facilitating the annotation of spatial transcriptomic data sets with cell-type labels. For instance, robust cell type decomposition (RCTD) [16] employs statistical models and maximum-likelihood estimation to infer cell-type proportions, whereas SpatialDWLS [17] applies damped weighted least squares for similar purposes. Seurat [18] utilizes a weighted nearest-neighbor approach to integrate data, while DestVI [19] and Cell2location [20] adopt a Bayesian hierarchical framework in conjunction with variational inference. In addition, deep-learning-based approaches such as Tangram [21] and Spatial-ID [22] have shown promise in processing spatial transcriptomic data with improved precision.

The brain is an exceptionally complex organ, characterized by heterogeneous cell types that vary significantly across different regions. One of the main challenges in understanding the neural circuits and mechanisms responsible for complex behaviors and neurological diseases is the lack of detailed spatial resolution in cell-type composition and gene-expression data. To address this, we have focused our efforts on applying a novel algorithm to brain tissue, aiming to uncover cellular heterogeneity with higher precision, and conducted preliminary tests on tumor samples to evaluate the broader applicability of the algorithm to other complex tissue types. As spatial omics data continue to expand rapidly, the computational demands and time-intensive nature of current methods pose significant barriers to their practical application. Although multiple approaches for mapping spatial transcriptomic data have been proposed, no method has yet achieved fine-grained cell-type mapping across multiple species and platforms. Herein, we introduce Deep Neural Network-based Spatial Cell Typing (DSCT)—a novel methodology that integrates a lightweight deep neural network model and an attention mechanism [23] to accurately infer detailed cell types from spatial transcriptomic data of the brain. Compared with other deep-learning methods, such as Tangram [21], which utilizes an optimized mapping strategy, or Spatial-ID [22], which employs a Graph Convolutional Network model, DSCT is built upon an efficient Multi-Layer Perceptron (MLP)-based neural network architecture. This design, enhanced by streamlined layers and residual connections, significantly improves the transmission of model parameters, expedites escape from local minima and improves accuracy by capturing complex, non-linear relationships in the data. The architectural advantages of DSCT result in significantly faster computational speed and reduced resource consumption compared with existing methods. Furthermore, its attention-based gene-selection mechanism effectively captures key gene-expression features, thereby reducing noise and enhancing model performance. As a result, DSCT provides highly accurate and resource-efficient computational solutions for spatial transcriptomics. Moreover, DSCT has demonstrated consistent accuracy and stability across multiple platforms, ensuring reliable results for various spatial transcriptomic data sets.

In this study, we evaluated the efficacy of DSCT across brain sections that were derived from different regions, species and spatial transcriptomic platforms. Results demonstrated that DSCT outperformed other computational integration methods in terms of accuracy, producing fine-grained cellular maps of diverse brain tissues. Furthermore, DSCT demonstrated remarkable processing speed and required minimal memory resources, enabling tasks that traditionally require high-performance computing resources to be completed on standard desktop computers. This advancement represents a robust, efficient and versatile approach to integrating single-cell multi-omics data, offering simplicity, speed and accuracy across diverse applications.

## RESULTS

### Workflow of DSCT

**Figure 1.**
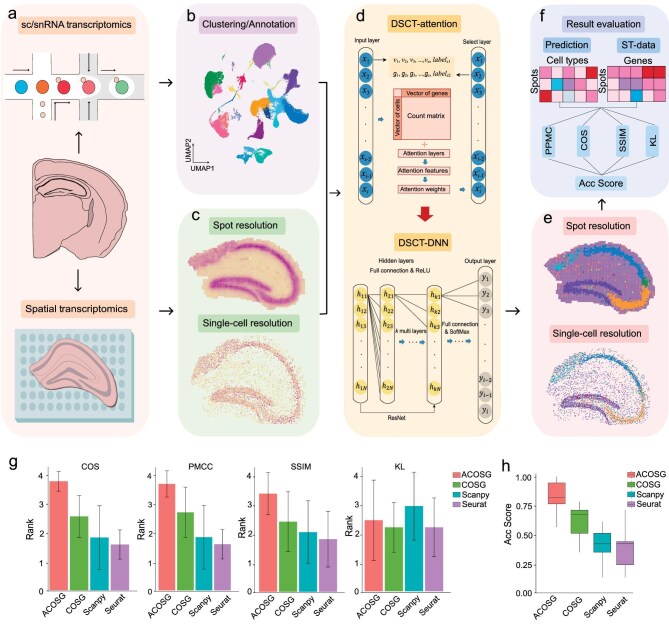
Deep Neural Network-based Spatial Cell Typing (DSCT) workflow. (a) Sample collection process, where top depicts collection of sc/snRNA-seq data and bottom shows collection of spatial transcriptomic data. (b) Clustering and annotation of sc/snRNA-seq data for utilization in DSCT processing with spatial transcriptomic data. (c) Spatial transcriptomic data acquired by Stereo-seq, showing distribution of gene density at different resolutions. (d) Schematic of DSCT training process, which first inputs sc/snRNA-seq and spatial transcriptomic data, then applies the attention mechanism to calculate attention weights, and sorts and filters feature genes according to weights. The filtered genes are then input into a deep neural network for training, with the trained model finally used to predict cell types in the spatial transcriptomic data. (e) Spatial cell-typing results acquired by DSCT at spot and single-cell resolutions. (f) Establishment of evaluation system integrating five distinct metrics: PPMC, COS, SSIM, KL and JS, designed to quantitatively assess the precision of spatial transcriptomic data predictions. ST, spatial transcriptomics; PPMC, Pearson correlation; COS, cosine similarity; SSIM, Structural Similarity Index; KL, Kullback–Leibler divergence. (g) Acc Score of different algorithms across the brain spatial transcriptomic data sets that we used (*n* = 9) through various mapping methods. Each box represents the interquartile range of the scores, with the line inside the box denoting the median. Whiskers extend from the minimum to the maximum values. (h) Rank of the metrics by using different gene-selection methods across the brain spatial transcriptomic data sets that we used (*n* = 9). The bars represent the average rank, with error bars indicating variability across different data sets.

Due to the complex and highly heterogeneous nature of the nervous system, annotating spatial cell types in brain spatial transcriptomic data presents a great challenge. Here, we introduce a comprehensive workflow for spatial cell typing, designated as DSCT (Fig. 1). Initially, DSCT requires inputting scRNA/single-nucleus (snRNA)-seq data annotated with cell-type information alongside raw spatial transcriptomic data (Fig. 1a–c). Subsequently, DSCT filters and retains genes that are co-expressed in both sc/snRNA-seq and spatial transcriptomic data sets. During this filtering process, an attention mechanism is applied to the selection of feature genes. By applying weights that are derived from training on labeled sc/snRNA-seq data with the attention mechanism, the importance of the feature genes is ranked, allowing their selective retention in sequence. Following this, the filtered sc/snRNA-seq data set is used to construct a sophisticated lightweight deep neural network model comprising five hidden layers. This model boasts a fully connected architecture with initial and terminal stage connections inspired by the ResNet [24] philosophy (Fig. 1d). This architectural choice facilitates not only uninterrupted information flow across the network, but also the ability to capture and leverage residual learning, thereby enhancing model performance and its ability to discover complex biological patterns precisely and efficiently. The cross-entropy loss function [25] is employed to compute loss during forward propagation and the Adagrad optimizer [26] is applied in backward propagation for calculating loss gradients and updating parameters. The application of this trained synergistic model to spatial transcriptomic data yields a probability distribution matrix for cell types within the data (Fig. 1e).

To evaluate the accuracy of the predicted distribution, a specialized multi-metric evaluation system is established by combining multiple similarity indices, including cosine similarity (COS) [27] to measure their directional similarity, Pearson product-moment correlation coefficient (PPMC) [28] to assess linear relationships, structural similarity (SSIM) [29] to evaluate the consistency of structural information and Kullback–Leibler Divergence (KL) [30] to quantify information loss (Fig. 1f). Then, the rankings of the results are averaged to serve as our accuracy score (Acc Score) [31,32]. This integrative approach enables a comprehensive and objective assessment of the spatial typing results that are obtained by using different computational methods.

As we all know, marker genes selection is critical for accurate spatial cell typing. There have already been many algorithms available for marker gene selection, including, Seurat [18], Scanpy [33] and COSG [34]. These traditional methods, especially Seurat and Scanpy, often tend to capture highly expressed feature genes, but they lack specificity and offer limited adaptability in parameter selection. In contrast, the COSG method demonstrates better capability in identifying feature genes with greater cell-type specificity. Additionally, the parameter settings in COSG provide the flexibility for adjustments to balance the specificity and expression of feature genes across cell types. To further adapt it for the neural network framework of our DSCT, we have enhanced COSG with an attention mechanism, which we called ACOSG. This integration aims to utilize a deep-learning perspective for more effective feature gene identification, which can help DSCT to accurately and efficiently recognize the spatial gene-expression features in the brain tissue.

To better evaluate the performance of ACOSG, we utilized previously reported typical data sets, including spatial transcriptomic data sets from (i) different platforms (Slide-seq [16], Stereo-seq [35], MERFISH [36], 10x Visium [37], STARmap [38]), (ii) different brain regions (hippocampus [16,35,37,38], cerebellar [35], olfactory bulb [35], cerebral cortex [36]), (iii) different species (human and mouse [36]) and corresponding sc/snRNA-seq data sets [35,39,40] for cell typing. In our results, ACOSG achieves higher precision in spatial cell typing, as evidenced by its better performance across multiple evaluation metrics compared with other gene-selection strategies (Fig. 1g and h). Specifically, we compared Acc Score, which combined four metrics across different gene-selection algorithms for all data sets. The box plot showed that the ACOSG demonstrated optimal performance compared with the others (Fig. 1g), indicating its notable effectiveness in gene selection across diverse data sets. Moreover, in separate metrics, our ACOSG method ranks first in the COS, PMCC and SSIM indices, and, in the KL index, it ranks second, showing overall strong performance (Fig. 1h).

### DSCT exhibits faster computing speed and requires fewer computing resources compared with other methods

**Figure 2.**
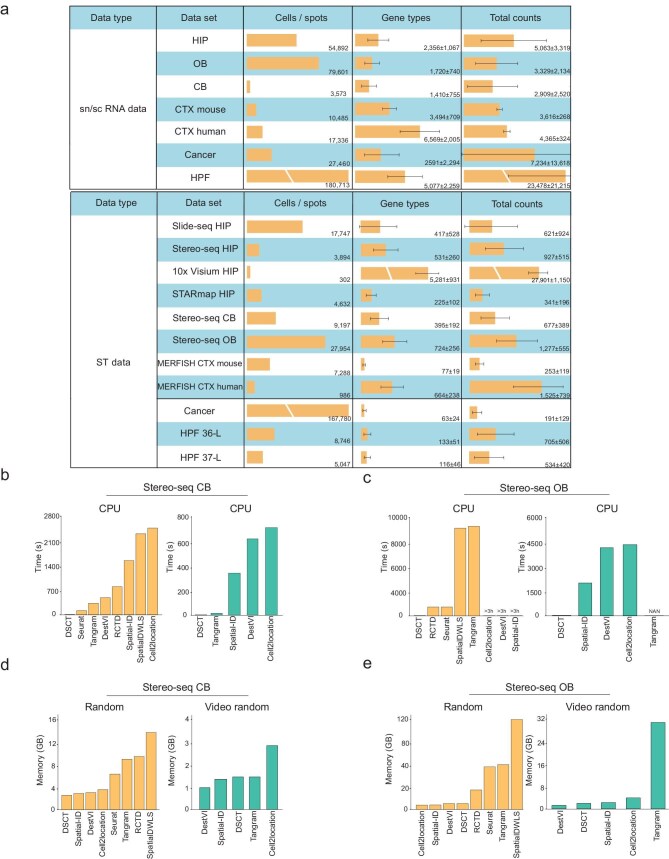
Higher computational and memory-usage efficiency of DSCT compared with other methods. (a) Number of cells/spots, genes and total counts in snRNA-seq data sets (top) and spatial transcriptomic data sets (bottom) used in this study. Numbers and bars in graphs represent means and error bars indicate standard deviations. ST, spatial transcriptomics. (b and c) CPU and GPU runtimes of different cell-type-prediction methods in the cerebellum and olfactory bulb data sets on the Stereo-seq platform. (d and e) Random memory and video random memory costs of different cell-type-prediction methods in the cerebellum and olfactory bulb data sets on the Stereo-seq platform.

Efficiency in terms of running time and memory usage is a critical consideration for bioinformaticians, particularly in the context of large data sets. The DSCT method demonstrated outstanding performance in these areas, which will be of significant benefit to researchers. To effectively demonstrate the results pertaining to running time and space performance, an in-depth analysis was conducted on the relatively small data set (CB) and large data set (OB) as examples (Fig. 2a). For the CB brain region, the scRNA-seq data contained 3573 cells and 6569 genes, and the spatial transcriptomic data contained 9197 cells and 21 275 genes. For the OB brain region, the scRNA-seq data contained 79 601 cells and 30 327 genes, and the spatial transcriptomic data contained 22 523 cells and 18 677 genes (Fig. 2a).

**Figure 3.**
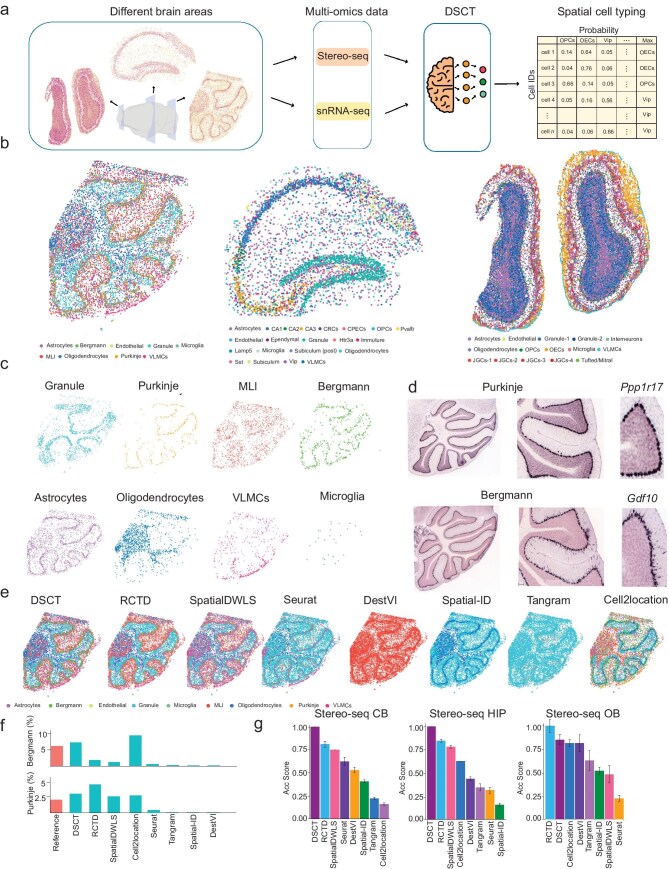
Superior performance of DSCT in spatial transcriptomic data sets across various brain regions. (a) Application of DSCT for spatial cell typing across different brain regions on the Stereo-seq platform. (b) DSCT-predicted spatial map of cell types in the cerebellum, hippocampus and olfactory bulb on the Stereo-seq platform. MLI, molecular layer interneuron; VLMCs, vascular leptomeningeal cells; CRCs, Cajal–Retzius cells; CPECs, choroid plexus epithelial cells; OPCs, oligodendrocyte precursor cells; JGCs, juxtaglomerular cells; OECs, olfactory ensheathing cells. (c) Spatial distribution of individual cerebellar cell types predicted by DSCT on the Stereo-seq platform. (d) Visualization of expression patterns of selected marker genes for Purkinje and Bergmann cell populations from the Allen Brain Atlas. (e) Comparison of cell-type predictions in cerebellar spatial transcriptomic data using various methods. (f) Comparison of percentages of Purkinje and Bergmann cells as predicted by different methods, with reference group illustrating the ratio found in the snRNA-seq data sets. (g) Calculation of accuracy (Acc) scores used to evaluate the precision of spatial cell typing in the cerebellum, hippocampus and olfactory bulb using different methods on the Stereo-seq platform.

Leveraging its neural network-based design, the DSCT method can exploit the acceleration potential of graphics processing units (GPUs). Here, results revealed that the processing speed of DSCT was much faster than that of other methods when accelerated by a GPU. Specifically, DSCT demonstrated remarkable efficiency in processing CB and OB data, completing predictions in an impressive 2.2 and 15.5 s, respectively (Fig. 2b and c). In contrast, although Tangram also demonstrated a rapid processing time for spatial cell-type prediction of CB data (15.6 s), it did not match DSCT in accuracy (Figs 2b, and 3e and g). For the OB data, DSCT completed predictions in 1% of the time required by other high-performance methods, such as DestVI (Figs 2c and 3g). Even in a central processing unit (CPU)-based environment, DSCT maintained remarkable efficiency, requiring only 3.8 s for CB data and 29.5 s for OB data (Fig. 2b and c), which is significantly faster than the time required by other methods, which ranged from 120 s for Seurat to >3 h for Spatial-ID (Fig. 2b and c). Despite its rapid execution of the CB data, Seurat did not yield impressive results in terms of accuracy (Figs 2b, and 3e and g). The running-time results for other data sets are shown in Table S1. Notably, the runtime of DSCT showed minimal variation between CPU and GPU environments, contrasting with other methods that are heavily reliant on GPU acceleration and exhibit marked increases in runtime on a CPU (Fig. 2b and c). This characteristic of DSCT emphasizes its reduced hardware dependency and greater adaptability, affirming its suitability for diverse computational settings.

Next, the memory and video memory usage of DSCT and other methods were compared while processing CB and OB data (Fig. 2d and e). Certain methods, such as SpatialDWLS and Tangram, far exceeded standard memory and video memory-usage capacities. For example, DSCT required only 8.2 GB of runtime memory, while SpatialDWLS required 123.4 GB to process OB data, which is far beyond the memory capacity of typical computers. Among the GPU-related methods, DSCT required only 1.9 GB of video memory to process OB data, while Tangram required 31.0 GB of runtime video memory, necessitating a server-level graphics card. The evaluation of space consumption for all methods (Table S2) confirmed that the efficiency of DSCT in hardware usage far exceeded that of other methods, thereby reducing the technical threshold in research processes. Notably, in all tests, DSCT operated smoothly on a standard home computer, indicating that researchers do not require cutting-edge laboratory equipment to perform accurate and rapid cell typing of spatial transcriptomic data.

### Application of DSCT to spatial transcriptomic data sets from different brain regions

The diversity of cell types organized in different brain regions ensures that each region can execute its specific functions. However, the specialization of cell types poses a challenge for consistent spatial cell typing across different brain regions. To assess its predictive accuracy and efficiency across diverse brain areas, we applied the DSCT model to previously published snRNA-seq and spatial transcriptomic data sets (Fig. S1) acquired using the 10x Genomics and Stereo-seq platforms, respectively [35]. Typical brain regions were selected, including the olfactory bulb (OB), hippocampus (HIP) and cerebellum (CB), which are characterized by distinct embryological origins and functional modules, encompassing a wide array of diverse cell types (Fig. 3a). Initially, the DSCT method was employed to generate a comprehensive spatial transcriptomic cell-type distribution matrix (Fig. 3a). This matrix was then mapped back to its original spatial loci, successfully reproducing the inherent spatial organization patterns of neuronal and non-neuronal cells (Fig. 3b). Notably, the DSCT model showed excellent performance compared with other widely reported algorithms, demonstrating exceptional functioning in both quantitative and qualitative dimensions (Fig. 3c–g).

Based on the prediction results that were obtained using the DSCT model, we successfully distinguished the tissue structure characterized by specific cell types in the three brain regions of interest (Fig. 3b). In the CB section, granule cells, molecular layer interneurons and Purkinje cells exhibited a distinct layered distribution (Fig. 3b and c). Among the non-neuronal cells, such as astrocytes (Astro), oligodendrocytes (Oligo), vascular leptomeningeal cells (VLMCs), Bergmann cells and microglia (Micro), Astro and Micro showed a more uniform distribution throughout the tissue whereas VLMCs and Oligo were primarily located in the outermost and innermost tissue layers, respectively (Fig. 3b and c). Remarkably, Bergmann cells were specifically distributed along the Purkinje layer, corroborating earlier reports [41]. In the HIP and OB sections, the DSCT predictions also revealed regional or layered distribution patterns of cell types (Fig. 3b). Specifically, within the HIP data set, distinct structures of the cornu ammonis (CA) regions and dentate gyrus (DG) were evident, especially CA2—a small structure that is located between CA1 and CA3 (Fig. S2a)—which was reminiscent of the expression of marker genes in the HIP (Fig. S2b). Similarly, in the OB, the DSCT predictions replicated the layered distribution characteristics of granule cells, tufted/mitral cells, glomerular layer cells and olfactory ensheathing cells (Fig. S2d and e). These observations align with established biological knowledge, reflecting the organizational patterns of cell types within tissues and emphasizing the accuracy of the DSCT method.

In evaluating cell-type-distribution accuracy, particular attention was paid to Purkinje and Bergmann cells within the CB region, given their specific concentration in the Purkinje layer within the CB (Fig. 3c). The *in situ* hybridization (ISH) results of cell-type markers that are available from the Allen Brain Atlas served as a reference (Fig. 3d). The arrangement of Bergmann cells was observed to be denser and more dispersed compared with that of Purkinje cells. This observation is consistent with the DSCT prediction results, providing indirect validation of its reliability and accuracy. For a more comprehensive comparison of accuracy within the CB region, several widely reported spatial cell-typing methods were selected for prediction and comparison (Fig. 3e). Results showed that the DSCT predictions more closely aligned with the biological characteristics of CB cell types, particularly in terms of the spatial distribution of Purkinje and Bergmann cells (Fig. 3e). An exploration was conducted to examine the proportions of Purkinje and Bergmann cells as predicted by various methods and compare these to the proportions of cell types observed in single-cell transcriptomic data (Fig. 3f). Findings revealed that the DSCT predictions most closely matched the cell-type ratio of Purkinje and Bergmann cells in the reference single-cell transcriptomic data, highlighting the precision of the DSCT method. Quantitatively, the highest accuracy (Acc) score achieved by the DSCT model in the CB data further underscored the accuracy of its spatial cell typing (Fig. 3g). Similarly, for assessing cell-type-prediction accuracy in the HIP and OB data sets, DSCT demonstrated superiority within the Acc Score quantitative evaluation framework (Fig. 3g) and exhibited more biologically plausible distribution patterns (Fig. S2c and f). This accuracy across different brain regions emphasizes the robustness of the DSCT method, further establishing its utility in cell-type prediction and spatial-distribution analysis.

In summary, the DSCT method demonstrated a high degree of stability and precision across various brain regions, affirming its reliability in cell-type identification and spatial-distribution prediction, and underscoring its considerable potential for intricate brain-tissue analysis.

### Application of DSCT to hippocampal spatial transcriptomic data sets from different platforms

**Figure 4.**
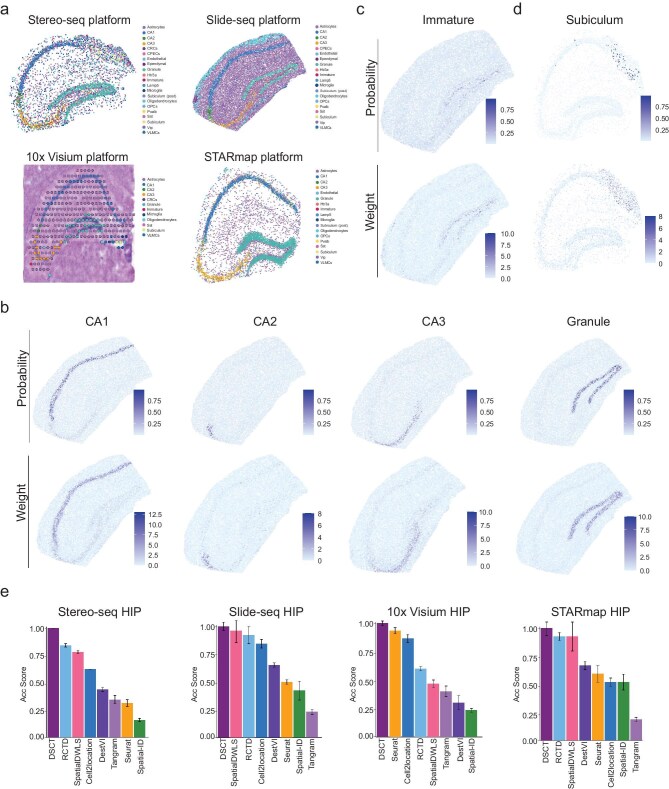
Superior performance of DSCT across different spatial sequencing platforms. (a) DSCT-predicted spatial map of cell types in the hippocampus on different platforms (Stereo-seq, Slide-seq, 10x Visium, STARmap). (b and c) Predicted spatial localization of cell types by DSCT for CA1, CA2, CA3, DG and immature neurons (top) and summed expression of top 20 cell-type-specific marker genes (bottom) on the Slide-seq platform. Color bar represents predicted cell-type proportion and gene-expression weight, respectively. (d) Predicted spatial localization of subiculum neurons by DSCT (top) and summed expression of top 20 specific marker genes for subiculum neurons (bottom) on the Stereo-seq platform. Color represents predicted cell-type proportion and gene-expression weight, respectively. (e) Calculation of Acc Score used to evaluate the precision of spatial cell typing in the hippocampus across different platforms (Stereo-seq, Slide-seq, 10x Visium, STARmap) using different methods.

The rapid development of spatial transcriptomic technology has led to the emergence of various spatial sequencing methods and corresponding platforms, posing major challenges to the universality and stability of spatial cell-typing methods across these diverse platforms. To ascertain the versatility and adaptability of the DSCT method across different platforms, we investigated four distinct technologies: Stereo-seq [35], Slide-seq [16], 10x Visium [37] and STARmap [10]. The HIP was selected as the focus area due to its typical distribution of cell types. Application of the DSCT model to the HIP data sets of each platform precisely mapped the territories of distinct cell types in each data set and clearly distinguished neuronal populations within the CA1, CA2, CA3 and DG regions (Fig. 4a). In the Stereo-seq, Slide-seq and STARmap platform data sets, which provide near-cellular resolution, DSCT distinguished finer GABAergic neuronal and non-neuronal cell types, such as *Pvalb*-, *Vip*- and *Htr3a*-positive GABAergic neurons and Astro. On the lower-resolution 10x Visium platform (generally containing 1–10 + cells per spot), DSCT reconstructed the spatial distribution of HIP cell types, consistently with the corresponding hematoxylin and eosin (H&E)-stained images (Fig. 4a).

To validate the spatial cell-typing accuracy of the DSCT method, its predicted distribution of various cell types was compared with the average expression distribution of marker genes. The initial focus was on CA and DG cells, comparing their predicted cell-type distribution and expression patterns of marker genes in the HIP (Fig. 4b). These marker genes were selected based on the top 20 differentially expressed genes for each cell type. A comprehensive comparison was performed across all platforms (Fig. 4b and Fig. S3a–c), revealing marked consistency between the predicted distribution of cell types and the expression patterns of marker genes across all platforms (Fig. 4b). The Slide-seq platform effectively captured enough immature neurons. The DSCT predictions indicated that these immature neurons were mainly distributed in the subgranular zone of the DG, consistently with marker gene-expression patterns of immature neurons in previous reports [42] (Fig. 4c). The subiculum was only observed in the Stereo-seq platform (Fig. 4a). The DSCT predictions indicated that the subiculum cells were precisely located on the subiculum structure, consistently with the spatial expression patterns of subiculum cell marker genes (Fig. 4d). Even on the 10x Visium platform with spot resolution, the DSCT method demonstrated excellent performance, providing precise and reliable predictions of cell-type distribution (Fig. S3c). These results collectively underscore the robustness and accuracy of the DSCT method across different platforms, even in the challenging context of spot-resolution analysis.

To compare the prediction accuracy of different spatial cell-typing methods, various algorithms were tested across four different platforms (Figs S2c and S3d–f). Evaluation of the prediction outcomes across all platforms revealed that DSCT not only accurately identified CA and DG cell types across all four platforms, but also accurately predicted other cell types such as GABAergic neurons and non-neuronal cells. Notably, DSCT was the sole method that was capable of precisely capturing CA and DG cells across all evaluated platforms. While the RCTD method also successfully predicted CA and DG cells across all platforms, it misidentified a high number of immature neurons, possibly mistaking some Astro as immature neurons. These findings suggest that DSCT is more effective at distinguishing cell types with similar gene-expression patterns. The previously established Acc Score criteria were further applied to quantitatively assess the performance of the different methods across the different platforms. Results showed that DSCT achieved the highest scores on all platforms (Fig. 4e). These data qualitatively and quantitatively confirmed the accuracy and stability of the DSCT algorithm across multiple platforms, highlighting its excellent recognition ability for both spot- and single-cell-resolution data sets.

### Application of DSCT to spatial transcriptomic data sets from different species

**Figure 5.**
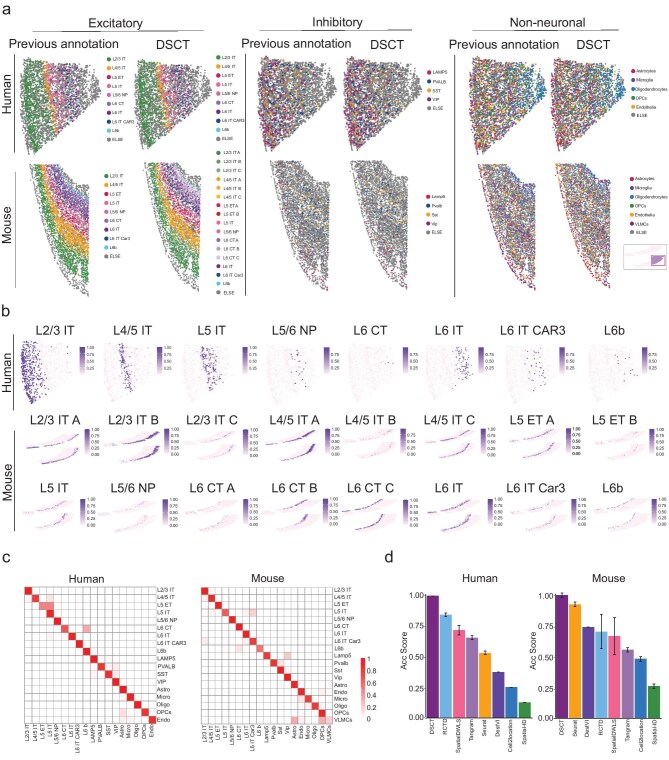
High accuracy of DSCT for cross-species analysis. (a) DSCT-predicted spatial map of cell types in the MERFISH human (top) and mouse (bottom) cortical spatial transcriptomic data for excitatory, inhibitory and non-neuronal cell groups. For each group, the left side represents previous annotated data and the right side represents DSCT prediction outcomes. Inset in bottom right corner highlights enlarged area in mouse cortex. (b) Predicted spatial localization of excitatory neuronal cell types by DSCT in the MERFISH human (top) and mouse (bottom) cortical spatial transcriptomic data. Color bar represents predicted cell-type proportion. (c) Confusion matrices of DSCT predictions for the MERFISH human and mouse cortical spatial transcriptomic data. Color represents overlap proportion of cell-type classification on *x*-axis (DSCT prediction) to cell-type classification on *y*-axis (previous annotated data). (d) Calculation of Acc Score used to evaluate the precision of spatial cell typing of the MERFISH human and mouse cortical data under different methods.

The variability in gene-expression complexity across different species poses a challenge, as methods that are effective in one species may not be suitable for others. To validate the applicability of the DSCT method across different species, we further tested human and mouse cortical tissues. Spatial transcriptomic data sets of human middle temporal gyrus (MTG) and mouse primary visual cortex (VIS), produced by the MERFISH platform [36], were selected, alongside previously reported corresponding single-cell transcriptomic data [39] for spatial cell typing (Fig. S4a-l). Prior to the prediction process, cortical cells were categorized into three major classes: excitatory neurons, inhibitory neurons and non-neuronal cells, to better illustrate the hierarchical distribution of different cell types in the cerebral cortex. The DSCT method was then used to map the spatial organization patterns of the excitatory neurons, inhibitory neurons and non-neuronal cells in the human and mouse cortices (Fig. 5a). Remarkably, the DSCT mapping results were highly consistent with the previous annotation from the MERFISH data sets (Fig. 5a). In the human and mouse data sets, DSCT accurately mapped the L2/3, L4, L5 and L6 intratelencephalic (IT) neurons, revealing a pronounced and similar stratification in both species. Distinctly, the L6b neurons presented divergent patterns: in the human MTG, these neurons were broadly dispersed across layer L6, even reaching into L5 and the adjacent white matter. Conversely, in the mouse VIS, L6b neurons constituted a more defined, narrow band at the lower boundary of layer L6. This observed divergence in L6b neuronal distribution is consistent with earlier studies and the MERFISH data (Fig. 5b). In the prediction of excitatory neuronal types within mouse data sets, refined scRNA-seq data sets with detailed annotations were utilized, allowing the division of IT and other excitatory neurons into distinct subtypes (Fig. 5a and b). The DSCT prediction results accurately located these subtypes within their specific cortical layers, demonstrating its ability to differentiate and identify more finely divided subtypes. Observations of inhibitory neuron and non-neuronal cell distributions in the human and mouse data also revealed biologically meaningful patterns. In humans, VIP neurons were mainly concentrated in the superficial layer of the cortex, while other types of inhibitory neurons exhibited more uniform distributions. Interestingly, in mice, both VIP and Lamp5 neurons were primarily distributed in the upper layer of the cortex, while other cell types exhibited more scattered distribution patterns (Fig. 5a and b). These results are consistent with previous findings [43] and reflect the essential characteristics of neuronal distribution, although the biological implications of these species-specific differences in the laminar organization of cortical neurons require further investigation.

Quantitative analysis was subsequently performed to compare the accuracy of cell-type predictions by various spatial cell-typing methods and previous annotated data. As expected, the DSCT-predicted cell types showed excellent correspondence to the previous annotated data, with an average accuracy rate of 95% for human data and 90% for mouse data. The only slight distributional discrepancy observed was for L6 CT neurons in the human cortex and VLMCs in the mouse cortex, where some cells were misidentified as L6b neurons and Astros, respectively (Fig. 5c). This prediction bias may be due to the scarcity of L6 corticothalamic neuron (CT) cells in the human cortical scRNA-seq data and the small number of total genes (only 242) in the mouse scRNA-seq data, which impacts training. Despite these limitations, the performance of DSCT was outstanding, ranking among the best of all methods tested (Fig. 5c and Fig. S5). Furthermore, Acc Scores were used to quantitatively evaluate the prediction results of the selected algorithms (Fig. 5d). The performance of DSCT ranked first for both human and mouse data. After comprehensive qualitative and quantitative comparison with other spatial typing methods, DSCT demonstrated exceptional stability and reliability in predicting cell types across different species with different biological complexity.

### Application of DSCT to finer cell-type annotation

To evaluate the performance of the DSCT algorithm in mapping more detailed cell types, we analysed the MERFISH data set [44], which contains comprehensive cell-type annotations at both the cluster and supertype levels. Specifically, we extracted spatial transcriptomic data of hippocampal tissue from 60 mouse brain slices, including 2 slices from the left hemisphere and 2 slices from the right hemisphere (Fig. S6a).

**Figure 6.**
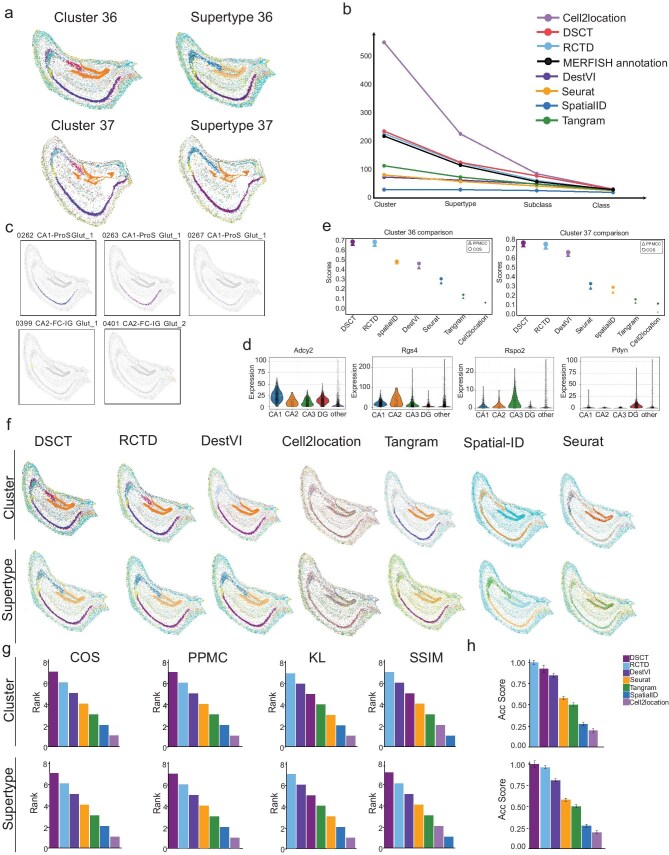
More refined cell-type classification of DSCT. (a) Distribution of hippocampal cell types at the cluster and supertype levels for data sets 36 and 37. (b) Number of cell types predicted by each algorithm at the cluster, supertype, subclass and class levels. (c) Distribution of cell types in the CA1 and CA2 regions. (d) Violin plot depicting average expression levels (*y*-axis) of key marker genes across cell types (*x*-axis) identified by DSCT. (e) Similarity metrics (PPMCC, COS) comparing cell proportions from all algorithms and original spatial transcriptomic data at the cluster level, with triangles representing PPMCC and circles representing COS. (f) Hippocampal cell-type prediction results of all algorithms at the cluster and supertype levels. (g) Scores of four metrics (COS, PPMCC, KL, SSIM) across all algorithms at the cluster and supertype levels. (h) Acc Score for all algorithms at the cluster and supertype levels.

Using the DSCT algorithm, we performed cell typing at both the cluster and supertype levels and validated the results by examining cell types and gene-distribution patterns. Notably, hippocampal structures, including the CA1, CA2, CA3 and DG structures, were mapped with higher granularity (Fig. 6a). Our analysis successfully defined 205 cluster types and 103 supertype types in data set 36 (C57BL6J-638850.36) and 179 cluster types and 100 supertype types in data set 37 (C57BL6J-638850.36) (Fig. 6a and b). The former annotated MERFISH data set identified 189 cluster types and 94 supertype types in data set 36 and 160 cluster types and 81 supertype types in data set 37, closely matching our refined classification (Fig. 6a and b, and Fig. S7a). Of note, the Cell2location algorithm, while reporting the highest number of cell types (Fig. 6b), incorrectly mapped several ineffective cell types from non-hippocampal regions, such as the cortex (CTX), within the hippocampus (Fig. 6f) and the expected distribution of cell types in the hippocampal region was not adequately captured (Fig. S6b). In contrast, our analyses successfully identified additional CA1 regional cell types at the cluster level, such as 0262 CA1-ProS Glut_1, 0263 CA1-ProS Glut_1 and 0267 CA1-ProS Glut_1 (Fig. 6c). Furthermore, while previous studies indicated a high degree of gene-expression similarity between the CA2 and fasciola cinerea, we successfully distinguished between these two cell groups, validating earlier findings (Fig. 6c). We also observed the expression of key marker genes (Adcy2, Rgs4, Rspo2 and Pdyn) that are characteristic of the CA1, CA2, CA3 and DG regions in the hippocampus. The expression patterns of these genes in different cell types confirmed the high accuracy of our predictions (Fig. 6d).

We conducted a comprehensive quantitative validation of the cell-typing results produced by DSCT at both the cluster and supertype levels to ensure accuracy. We compared the similarity between the cell-type proportions generated by the DSCT algorithm and the original annotations. Using the COS and Pearson product-moment correlation coefficient (PPMCC) indicators, DSCT demonstrated the highest similarity, confirming the robustness of its mapping results (Fig. 6e). To further assess cell-typing performance, we applied multiple algorithms (RCTD, DestVI, Cell2location, Tangram, Spatial-ID and Seurat) in repeated cell-typing experiments. Notably, DSCT performed well at both the supertype and cluster levels, particularly in the context of biological hierarchical observations (Fig. 6f). In terms of quantitative metrics, DSCT outperformed other algorithms, achieving superior PPMCC and COS scores—metrics commonly used to measure correlation—across both cluster and supertype levels (Fig. 6g). Additionally, DSCT demonstrated high performance in the Acc Score metric, which provides a comprehensive evaluation of classification accuracy (Fig. 6h). Notably, our model significantly improved processing efficiency without compromising classification accuracy. DSCT completed the cell-typing task in just 27.1 s for the cluster level and 16.3 s for the supertype level. In comparison, the similarly precise RCTD method required 18.9 and 2.7 h, respectively, to process the same data (Table S1). This substantial enhancement in efficiency is crucial for handling large-scale transcriptomic data sets.

## DISCUSSION

The computational integration of spatially resolved transcriptomic and scRNA-seq data combines the advantages of both technologies and overcomes the inherent shortcomings of spatial transcriptomics, such as low throughput and limited capture rates. However, existing methods often demand extensive computing resources and time, and their accuracy and versatility across different platforms and species remain uncertain. In this context, the proposed DSCT algorithm demonstrates excellent spatial cell-typing ability in different brain regions, platforms, species and finer cell types. This approach not only demonstrates higher accuracy and scalability compared with existing methods, but also requires fewer computing resources and time.

DSCT is a predictive method that relies on the specificity and depth of gene capture (*in situ* capture technique) and the specificity and quantity of probe selection (*in situ* sequencing and *in situ* hybridization techniques). In our analysis, we observed obvious error bars in the evaluation of spatial cell typing when using STARmap (*in situ* sequencing technique) data sets (Fig. 3g), indicating substantial variability in the Acc Score metrics. This variability may stem from probe selections that do not effectively capture gene features, as current probes are generally designed for the entire brain rather than specific brain regions. Additionally, the *in situ* sequencing technique may bring considerable background noise, affecting data clarity. Therefore, it is advisable to optimize probe designs by customizing them for specific brain regions, in order to better capture spatial gene-expression characteristics and improve prediction accuracy.

Previous studies have typically used matched sc/snRNA-seq data from adjacent tissue sections to perform spatial cell typing [21]. Our results demonstrated that single-cell and spatial transcriptomic data from non-identical tissue can also be effectively integrated (Figs 4 and 5), validating the robustness of the DSCT method. Nevertheless, using sc/snRNA-seq data from adjacent tissue sections enhanced spatial cell-typing accuracy. In situations in which scRNA data included cells from the subiculum, but the spatial data sets of Slide-seq and STARmap lacked this structural representation, inaccuracies arose in spatial mapping, leading to the incorrect identification of some cells as subiculum cells (Fig. 4a and d). Consequently, employing sc/snRNA-seq data from tissue sections that are adjacent to those under study for spatial cell-type annotation by DSCT tended to yield more accurate results.

Although this study primarily focused on the application of DSCT to brain tissue, the underlying theoretical framework also makes it well suited for use in other complex biological tissues, such as tumors. DSCT has the capability to identify spatially resolved heterogeneous cell populations within tumor microenvironments, offering valuable insights into the cellular architecture of tumors. We analysed the human breast cancer data set and successfully reproduced the distinct distribution patterns of two ductal tumors, as well as the expected distribution of diverse cell types within the same tumor tissue (Fig. S8a). These findings are consistent with well-established biological characteristics of tumors [45]. In addition, our algorithm demonstrated the highest accuracy when compared with the original spatial transcriptomic annotations (Fig. S8b). To further validate the accuracy of our classification, we compared the gene-expression profiles of key marker genes (*FGL2, POSTN, BANK1, TCIM, FASN, IL7R, KRT6B* and *CEACAM6*) with our classifications, with the expression patterns showing strong concordance (Fig. S8c and d). We also explored the biological functions of CD8+T cells and invasive tumor cells based on the classification results that were generated by DSCT (Fig. S8e). Results revealed a strong correlation between the classified cell types and their known cellular functions. Specifically, CD8+T cells exhibited notable activity in immune surveillance and tumor suppression, while invasive tumor cells displayed enhanced cell proliferation and migration, associated with elevated expression of invasive markers and extracellular matrix interacting molecules. This detailed characterization of cellular functions not only provides deeper insights into the cell–cell interactions and communication networks within a tumor microenvironment, but also offers potential pathways for identifying novel therapeutic targets. In addition, the applicability of DSCT extends beyond transcriptomics. It can be adapted for use in other omics fields, such as proteomics and epigenomics, providing a more comprehensive understanding of biological processes at the subcellular level. This flexibility positions DSCT as a versatile tool for studying a wide range of biological systems.

When discussing the potential limitations of the DSCT method, we should focus on two core issues. Firstly, due to technical differences and inconsistent sequencing depths between different spatial transcriptome platforms (such as Stereo-seq, STARmap, Slide-seq), data noise may affect the accurate interpretation of spatial gene information by mapping algorithms. Secondly, the effectiveness of the DSCT algorithm is highly dependent on the accuracy of single-cell annotation and inaccurate annotations may have a negative impact on the final results. To overcome these limitations, we plan to utilize spatial continuity and intercellular relationships in future research to reduce data noise and explore the development of more accurate and targeted automatic single-cell transcriptome annotation algorithms that are currently lacking. Although DSCT has room for improvement in certain areas, it demonstrates outstanding performance in cell-type annotation across species, platforms, various brain regions and fine-grained cell types. It outperforms other methods by achieving high accuracy with low computational resource demands and fast processing speeds. This method provides an efficient, reliable tool and a new perspective for spatial transcriptomic research, which is anticipated to further propel advancements and innovation in the field.

## METHODS

In this study, we systematically collected published spatial transcriptomic data sets from various platforms, encompassing multiple brain regions. In detail, cerebellar and OB data sets were obtained from the Stereo-seq platform, while hippocampal data sets were sourced from the Stereo-seq, 10x Visium, 10x Xenium, STARmap and Slide-seq platforms. For cortical data, human MTG and mouse primary visual cortex data sets were obtained from the MERFISH platform. All these data sets are publicly available, as shown in the ‘Data availability’ section. Notably, the spatial transcriptomic annotations that were derived from the MERFISH platform were used as the previous annotated reference for validating and comparing our results. Concurrently, previously published sc/snRNA-seq data that were relevant to these brain regions were collated and annotated to facilitate subsequent cell typing. Detailed materials and methods are available in the Supplementary data.

## Supplementary Material

nwaf030_Supplemental_Files

## Data Availability

Various spatial transcriptomic and sc/snRNA-seq data sets that are available from public repositories were used in this study. For spatial transcriptomics, Stereo-seq data sets of the cerebellum, hippocampus and OB were accessed from the Brain Data Center (https://www.braindatacenter.cn/datacenter/web/#/dataSet/details?id=1712760331765706754). Slide-seq hippocampal data were obtained from the Broad Institute Single Cell Portal (https://singlecell.broadinstitute.org/single_cell/study/SCP948/robust-decomposition-of-cell-type-mixtures-in-spatial-transcriptomics). Additionally, hippocampal data sets using 10x Genomics technology were obtained from 10x Genomics (https://www.10xgenomics.com/resources/datasets/adult-mouse-brain-coronal-section-fresh-frozen-1-standard), while STARmap hippocampal data were obtained from Zenodo (https://zenodo.org/records/8041114). The MERFISH human and mouse cortex data sets were obtained from datadryad (https://datadryad.org/stash/dataset/doi:10.5061/dryad.x3ffbg7mw). The acquisition of MERFISH hippocampal tissue is performed by Allen Institute (https://alleninstitute.github.io/abc_atlas_access/descriptions/MERFISH-C57BL6J-638850.html). For sc/snRNA-seq, cerebellar, hippocampal and OB data sets were sourced from the Brain Data Center (https://www.braindatacenter.cn/datacenter/web/#/dataSet/details?id=1712760331765706754), while human cortex data were obtained from the Allen Sea AD Atlas (https://registry.opendata.aws/allen-sea-ad-atlas/) and mouse cortex data (visual cortex) were obtained from the NCBI GEO database under GEO-GSE190940 (https://www.ncbi.nlm.nih.gov/geo/query/acc.cgi?acc=GSE190940). The acquisition of single-cell data corresponding to MERFISH hippocampal tissue is performed by Allen Institute (https://alleninstitute.github.io/abc_atlas_access/descriptions/WMB-10Xv2.html). The single-cell and spatial transcriptome data for cancer tissue were obtained from 10x Genomics (https://www.10xgenomics.com/products/xenium-in-situ/preview-dataset-human-breast). The code used in this study is openly available and can be accessed at the following GitHub repository: https://github.com/coffeei1i/DSCT. Additionally, a tutorial for setting up the required environment on CodeOcean is available at: https://codeocean.com/capsule/6490820/tree.
