# Supplementary material for: DSCT: a novel deep-learning framework for rapid and accurate spatial transcriptomic cell typing
Source: Natl Sci Rev. 2025 Jan 28;12(5):nwaf030. doi: 10.1093/nsr/nwaf030 (PMC12045154; doi:10.1093/nsr/nwaf030)
Supplement: nwaf030_Supplemental_Files [file nwaf030_supplemental_files.zip › Supplementary Figure.pdf]

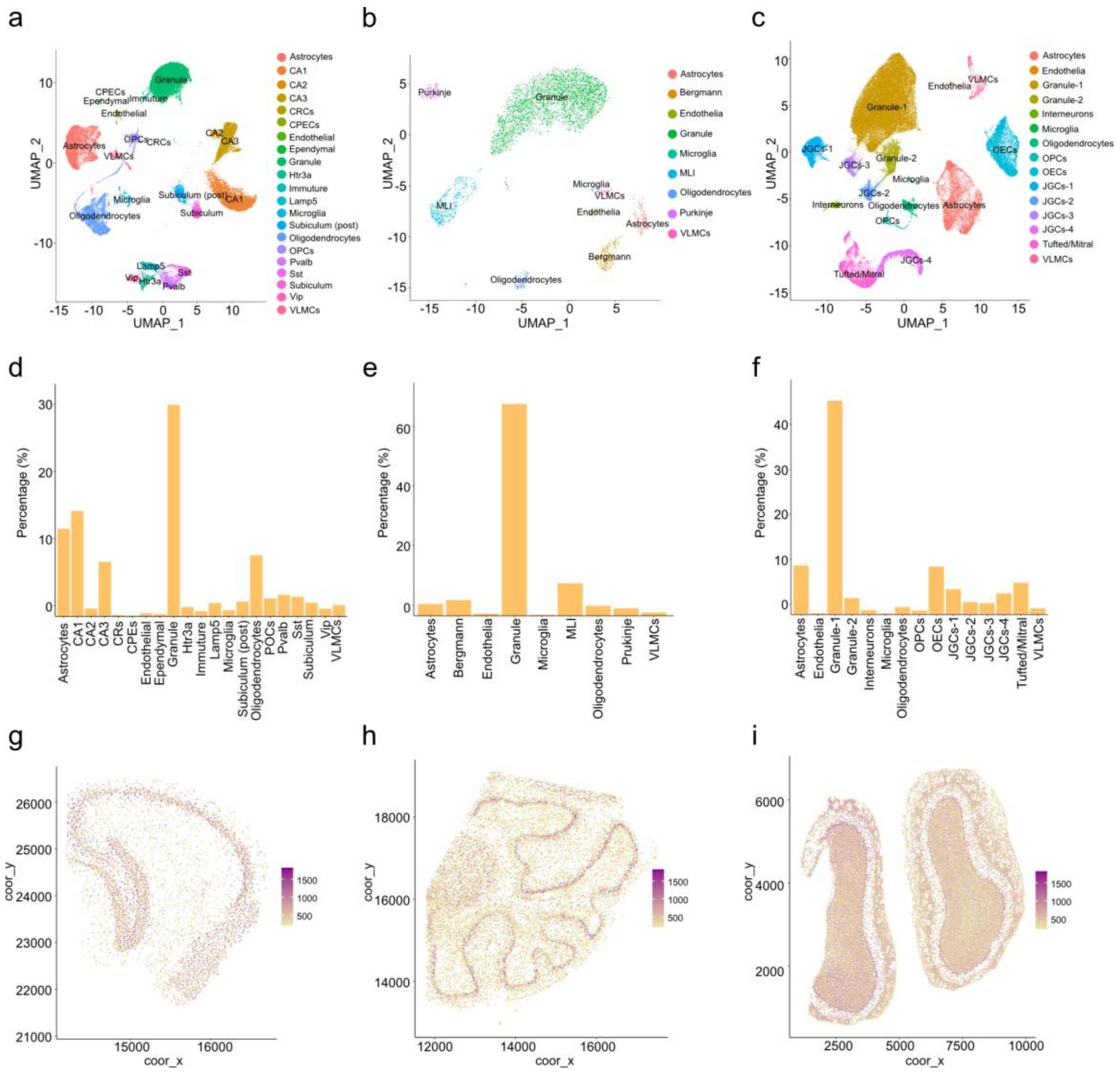

Supplementary Fig. 1 | SnRNA-seq and spatial transcriptomic datasets of mouse hippocampus, cerebellum, and olfactory bulb.

a-c, Uniform Manifold Approximation and Projection (UMAP) embedding plots of hippocampus, cerebellum, and olfactory bulb snRNA-seq data, with nuclei colored by cell clusters. VLMCs vascular leptomeningeal cells; CRCs, Cajal-Retzius cells; CPECs, choroid plexus epithelial cells; OPCs, oligodendrocyte precursor cells; MLI, molecular layer interneuron; JGCs, juxtaglomerular cells; OECs, olfactory ensheathing cells.

d-f, Proportion of cell types in the hippocampus, cerebellum, and olfactory bulb snRNA-seq data.

g-i, Total counts distribution of hippocampus, cerebellum, and olfactory bulb spatial transcriptomic data from the Stereo-seq platform. The color bar represents the number of counts, ranging from light yellow to dark purple from low to high.

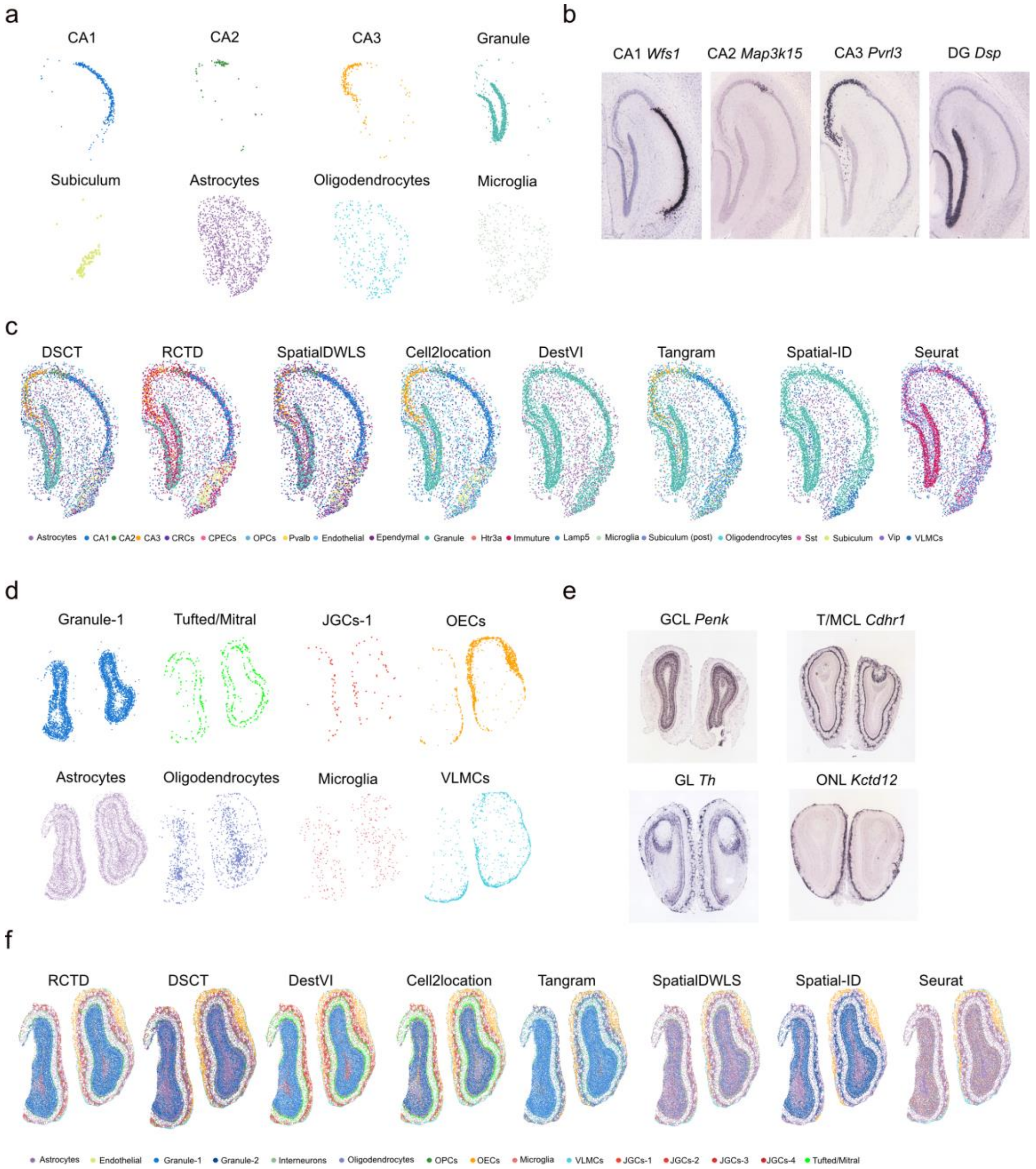

Supplementary Fig. 2 | Application of DSCT and control methods to mouse hippocampal and olfactory bulb datasets using the Stereo-seq platform.

a, Distribution of individual hippocampal cell types predicted by DSCT on the Stereo-seq platform. VLMCs vascular leptomeningeal cells; CRCs, Cajal-Retzius cells; CPECs, choroid plexus epithelial cells; OPCs, oligodendrocyte precursor cells;

b, Expression patterns of selected marker genes for CA1 (*Wfs1*), CA2 (*Map3k15*), CA3 (*Pvrl3*), and DG (*Dsp*) cell populations from the Allen Brain Atlas.

c, Cell-type prediction in hippocampal spatial transcriptomic data using various methods.

d, Distribution of individual olfactory bulb cell types predicted by DSCT on the Stereo-seq platform. JGCs, juxtaglomerular cells; OECs, olfactory ensheathing cells.

e, Expression patterns of selected marker genes for GCL (*Penk*), T/MCL (*Cdhr1*), GL (*Th*), and ONL (*Kctd12*) cell populations from the Allen Brain Atlas. GCL, granule cell layer; T/MCL tufted/Mitral cell layer; GL, glomerular layer; ONL, olfactory nerve layer.

f, Cell-type prediction in olfactory bulb spatial transcriptomic data using various methods.

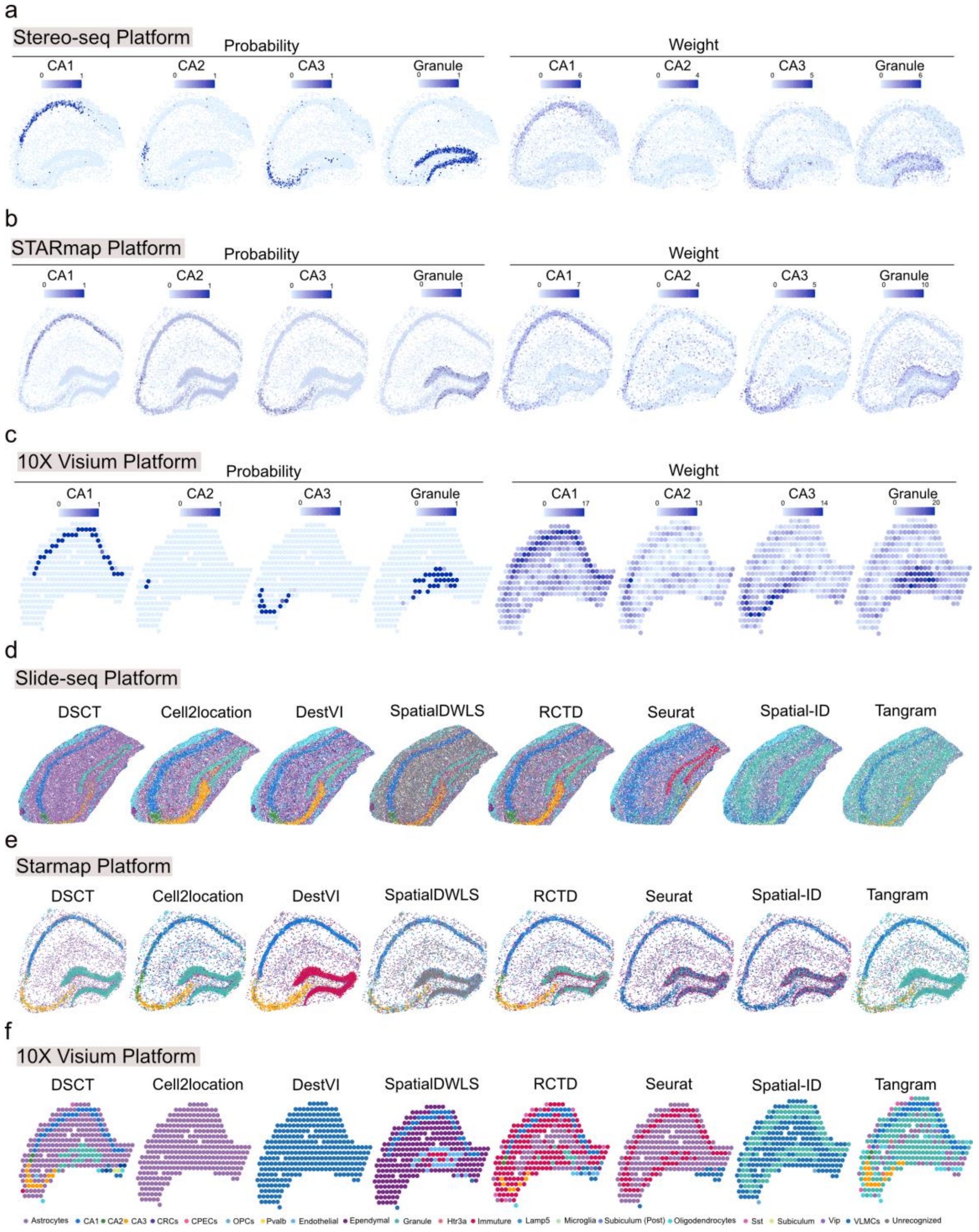

Supplementary Fig. 3 | Application of DSCT and control methods to mouse hippocampal datasets using different platforms.

a-c, Probability distribution of predicted spatial localization of cell types by DSCT for CA1, CA2, CA3, DG neurons (probability), and summed expression of top 20 cell-type-specific marker genes (weight) on different platforms (Stereo-seq, STARmap, 10x Visium). Color bar represents predicted cell-type proportion and gene expression weight, respectively.

d-f, Cell-type prediction in hippocampal spatial transcriptomic data using various methods on different platforms (Slide-seq, STARmap, 10x Visium).

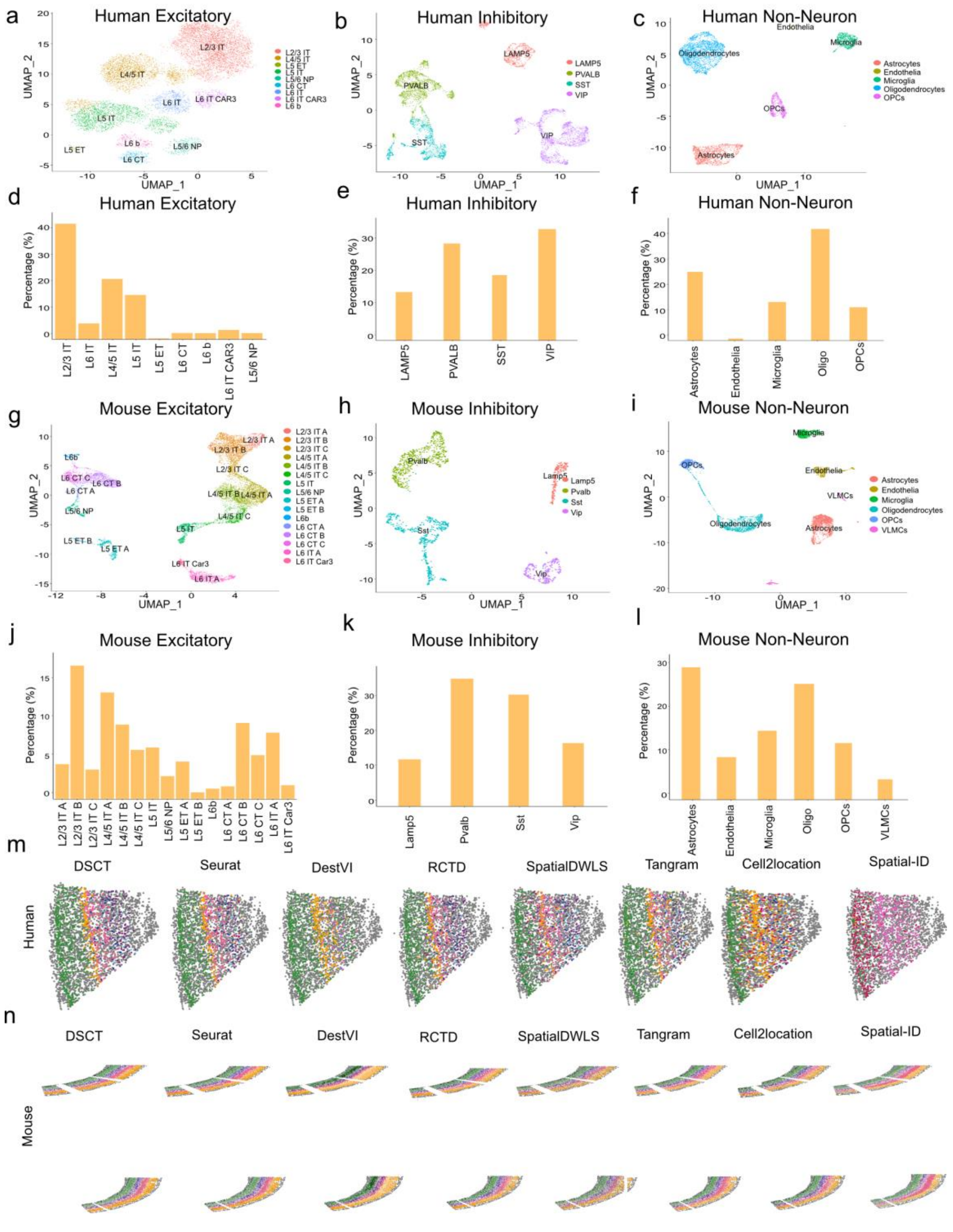

Supplementary Fig. 4 | SnRNA-seq datasets of human and mouse cortices using the 10x Genomics platform.

a-c, UMAP embedding plots of human cortical snRNA-seq data for excitatory, inhibitory, and non-neuronal cells, with nuclei colored by cell clusters. OPCs (Oligodendrocyte Precursor Cells).

d-f, Proportion of excitatory, inhibitory, and non-neuronal cell types in each human snRNA-seq dataset.

g-i, UMAP embedding plots of mouse cortical snRNA-seq data for excitatory, inhibitory, and non-neuronal cells, with nuclei colored by cell clusters.

j-l, Proportion of excitatory, inhibitory, and non-neuronal cell types in each mouse snRNA-seq

dataset.

m-n, Cell-type prediction in human and mouse cortex spatial transcriptomic data using various methods on different platforms.

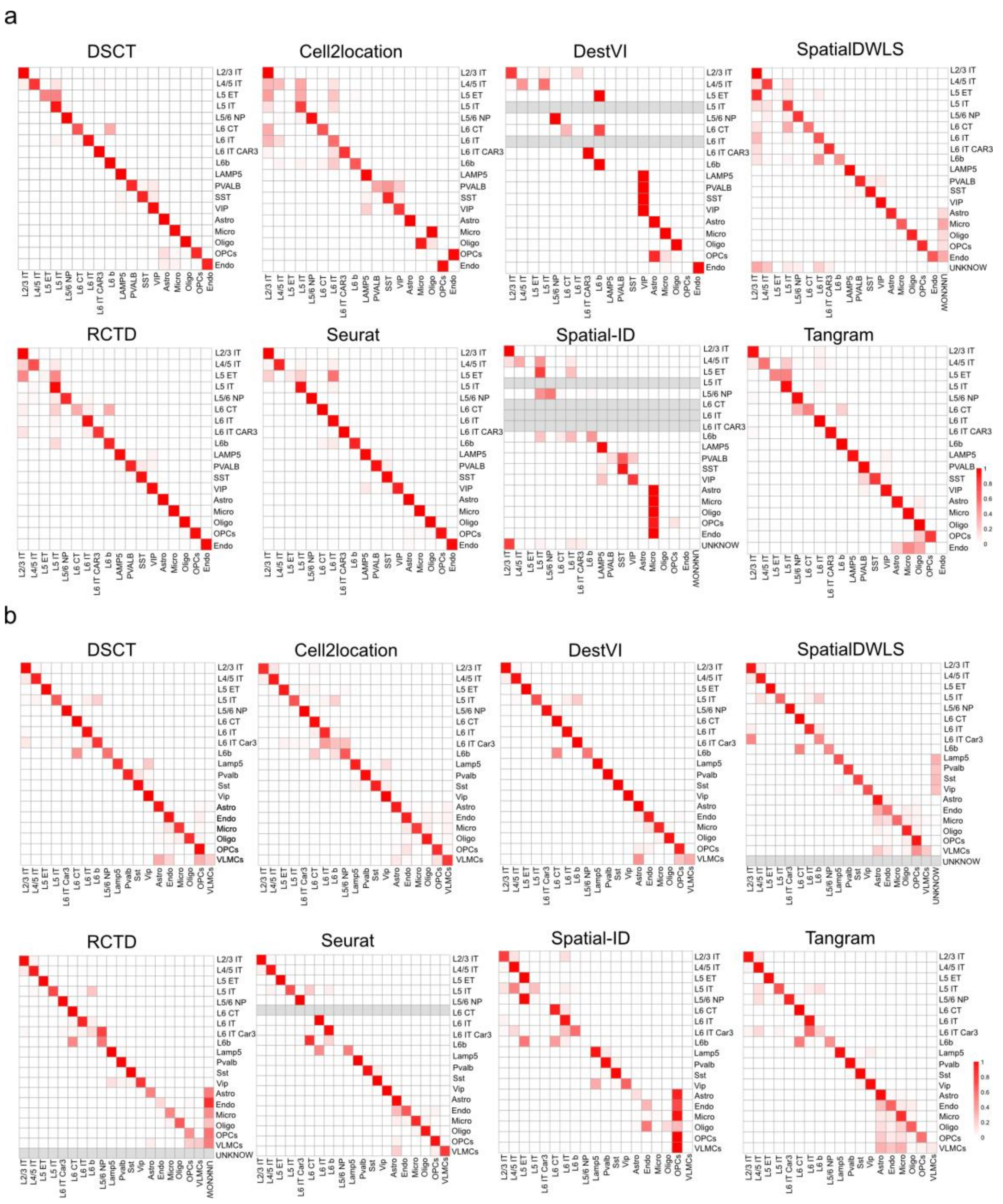

Supplementary Fig. 5 | Application of DSCT and control methods to human and mouse cortical spatial transcriptomic datasets using MERFISH.

a, Confusion matrices of different prediction methods for MERFISH human cortical spatial transcriptomic data. Color represents overlap proportion of cell-type classification on x-axis (prediction of chosen method) to cell-type classification on y-axis (former annotation).

b, Confusion matrices of different prediction methods for MERFISH mouse cortical spatial transcriptomic data. Color represents overlap proportion of cell-type classification on x-axis (prediction of chosen method) to cell-type classification on y-axis (former annotation).

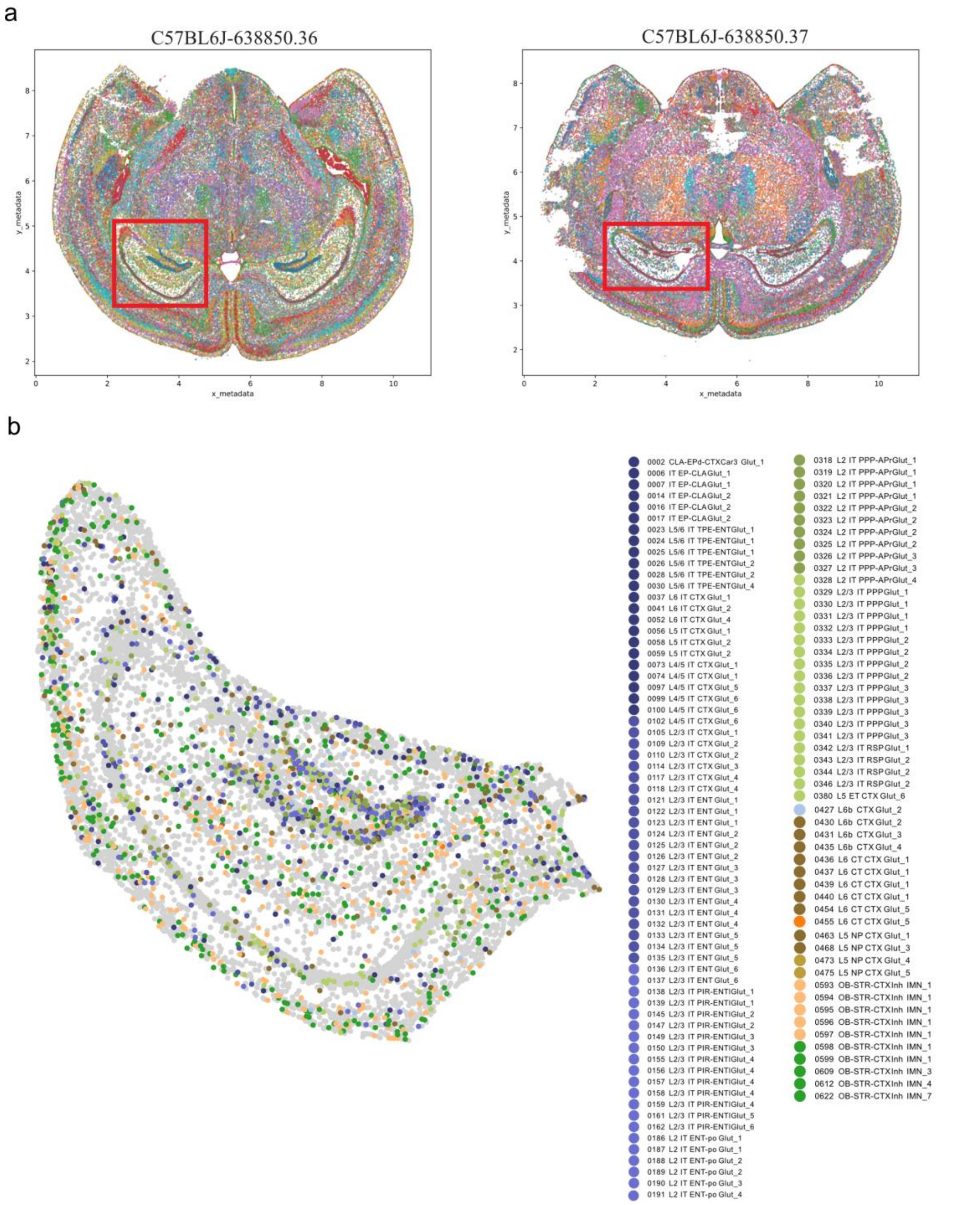

Supplementary Fig. 6 | Application of Cell2location to mouse hippocampal spatial transcriptomic datasets using MERFISH.

a, Hippocampal tissues from two selected coronal slices of the mouse brain. **b**, Distribution of non-hippocampal cell types identified in Cell2location. Of note, Cell2location recognized a more diverse range of cell types, but incorrectly assigned cell types originating from regions outside the hippocampus

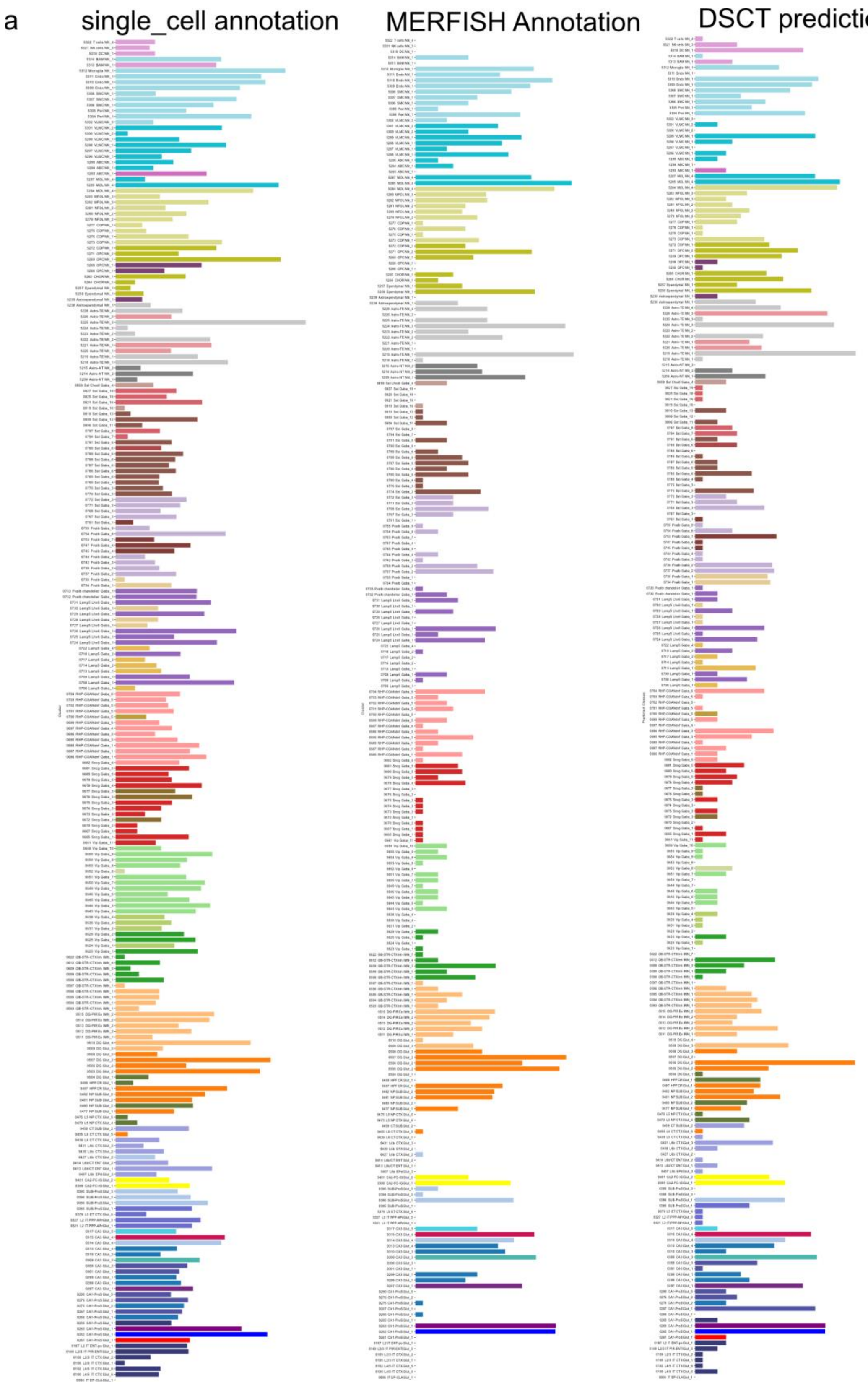

Supplementary Fig. 7 | Application of DSCT to mouse hippocampal spatial transcriptomic datasets using MERFISH.

a, Cell proportion distribution of original single-cell transcriptomic annotations, spatial transcriptomic annotations, and predicted spatial transcriptomic annotations.

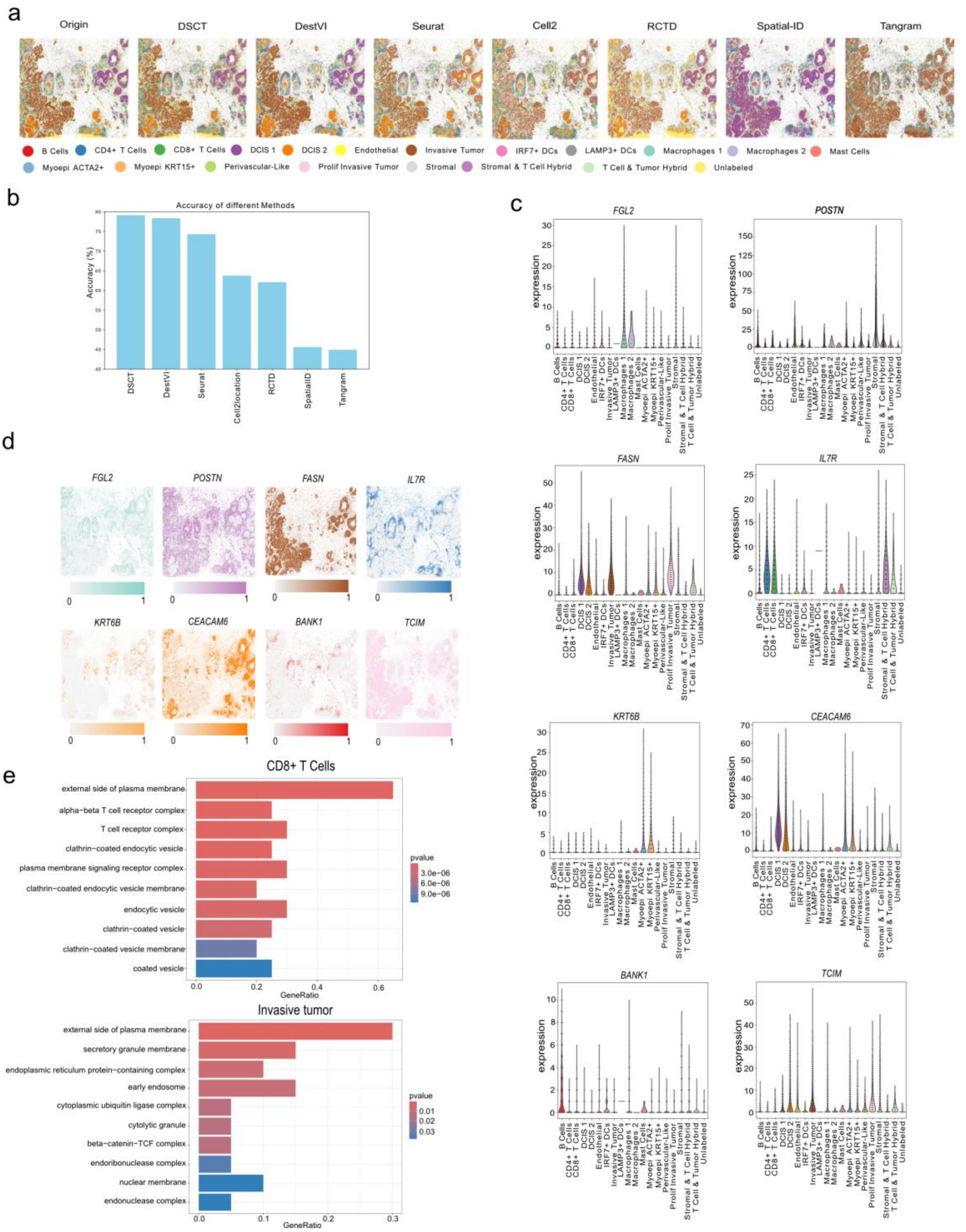

Supplementary Fig. 8 | Application of DSCT to human breast tumor dataset.

a, Cell-type predictions in breast tumor spatial transcriptomic data using various methods (Origin means former spatial transcriptomic annotation). **b**, Comparison of accuracy of all algorithm predictions with previous spatial transcriptomic annotations. **c**, Violin plot depicting average expression levels (y-axis) of key marker genes (*FGL2*, POSTN, *BANK1*, *TCIM*, *FASN*, *IL7R*, *KRT6B*, and *CEACAM6*) in cell types (x-axis) identified by DSCT. **d,** Distribution of key marker genes (*FGL2*, POSTN, *BANK1*, *TCIM*, *FASN*, *IL7R*, *KRT6B*, and *CEACAM6*) in tumor tissue. **e**, Pathways identified through GO analysis derived from top gene sets enriched in specific cell types (CD8+T cells, invasive tumors) identified by DSCT.

	Parameters	Value	Explanation
Data preprocess	sc_min_counts	0	Minimum number of counts required for single-cell data to be retained.
	st_min_counts	0	Minimum number of counts required for spatial data to be retained.
	sc_min_cells	20	Minimum number of cells in which a gene must be expressed to be retained in the single-cell data.
	st_min_cells	20	Minimum number of cells in which a gene must be expressed to be retained in the spatial transcriptomic data.
Marker gene selection	expressed_pct	0.1	Minimum percentage of cells in which a gene must be expressed.
	n_genes_user	30-60	Number of genes per cell considered for the calculation.
	n_top_genes	10000	Number of top highly variable genes to be retained.
Attention model	hidden_size	256	Number of neurons in the hidden layer.
	learning rate	0.01	Learning rate for the optimizer.
	loss function	CrossEntropyLoss	Loss function used during training.
	linear layers	3	Linear layers used to transform data through the network.
	Softmax layer	1	Softmax layer for normalizing attention scores.
	ReLU layers	1	ReLU activation layer for introducing non-linearity.
DSCT model	hidden_size1	256	Number of neurons in first hidden layer.
	hidden_size2	128	Number of neurons in second hidden layer.
	hidden_size3	64	Number of neurons in third hidden layer.
	hidden_size4	32	Number of neurons in fourth hidden layer.
	learning_rate	0.01	Learning rate for the optimizer.
	num_epochs	200	Number of epochs for which the model is trained.
	optimizer	Adagrad	Optimizer used for training.
	criterion	CrossEntropyLoss	Loss function used during training.
	ReLU layers	4	ReLU activation layer for introducing non-linearity.
	linear layers	5	Linear layers used to transform data through the network.

Supplementary Fig. 9 | Parameters for the DSCT algorithm.
