# Supplementary material for: DSCT: a novel deep-learning framework for rapid and accurate spatial transcriptomic cell typing
Source: Natl Sci Rev. 2025 Jan 28;12(5):nwaf030. doi: 10.1093/nsr/nwaf030 (PMC12045154; doi:10.1093/nsr/nwaf030)
Supplement: nwaf030_Supplemental_Files [file nwaf030_supplemental_files.zip › Supplementary Meterials and Methods.docx]

**Methods**

**Annotation of single-cell transcriptomic data**

The single-cell transcriptomic datasets utilized in this study included fully annotated datasets from published sources (CTX mouse, CTX human, Cancer, HPF MERFISH) and non-annotated datasets at the time of acquisition (HIP Stereo-seq, OB, CB). For the latter, manual annotation was performed to ensure accuracy and usability of the data. To effectively annotate the sc/snRNA-seq data as a reference for spatial transcriptomics, a comprehensive processing workflow was employed using the ‘Seurat’ package in R. Initially, rigorous quality control procedures were implemented to filter low-quality cells, followed by normalization of the filtered data to mitigate biases attributable to technical variations. Subsequently, unsupervised clustering was performed based on transcriptional characteristics to identify distinct cell populations. After clustering analysis, a manual review was conducted to exclude any clusters that may be influenced by external factors, further enhancing the accuracy and reliability of the data. Finally, each cell type of single-cell transcriptomic for training was annotated based on canonical markers. Using these procedures, each cell cluster was precisely categorized, providing a highly accurate and detailed single-cell transcriptomic reference database for spatial cell typing.

**Attention mechanism for feature gene selection**

The attention mechanism of the transformer framework can be utilized to identify key feature genes in gene expression data. Due to its unique capability to capture and analyze complex gene relationships in high-dimensional gene data, this mechanism provides a new perspective for mapping scRNA-seq and spatial transcriptomic data. Here, the input data are initially processed to normalize the single-cell gene expression matrix, with each row representing a cell and each column representing a gene. Genes detected in both the scRNA-seq and spatial transcriptomic datasets are identified through intersection, ensuring the unification and alignment of gene sets. The attention mechanism is used to capture and interpret complex interactions among genes. For calculating attention scores, a common gene expression matrix, denoted as $X\in R^{n\times d}$, where $n$ represents the number of cells and $d$ represents the number of genes, is established. Through linear transformation by the attention layer, the attention score of each gene can be calculated as:

$$A=Softmax\left( XW^{\left( atten \right)} \right)$$

where $W^{(atten)}$ is the learned weight matrix and the $Softmax$ function ensures that the output is positive and sums to 1, representing a probability distribution. Next, the attention scores are used to weight each feature in the input matrix $X$:

$$H=A\odot X$$

$H\in R^{n\times d}$ is the weighted feature representation of each cell, where $\odot$ represents element-wise multiplication. The weighted feature $H$ is then mapped to a hidden space to capture deeper feature relationships:

$$Z=RELU\left( HW^{\left( hid \right)}+b^{\left( hid \right)} \right)$$

where $W^{(hid)}\in R^{d\times h}$ and $b^{(hid)}\in R^{h}$ are the weights and biases of the hidden layer, $h$ is the dimension of the hidden layer, and $RELU$ is the activation function. Finally, the hidden layer output $Z$ is further mapped to the output space for classification tasks:

$$O=ZW^{(out)}+b^{(out)}$$

where $W^{(out)}\in R^{h\times c}$ and $b^{(out)}\in R^{c}$ are the weights and biases of the output layer and $c$ represents the number of categories. The model training process follows these steps, with cross-entropy loss $\mathcal{L}$ used to calculate the difference between model outputs $O$ and true labels $Y$:

$$\mathcal{L}\left( O,Y \right)=-\sum_{i=1}^{n} \sum_{j=1}^{c} Y_{ij}\log\left( O_{ij} \right)$$

During this process, the Adagrad optimizer is utilized to update the model weights, minimizing loss $\mathcal{L}$. After completion of training for the attention model, the information in the output matrix $O$ is used to select feature genes. By comparing the output vectors of different cell types, we can identify feature genes crucial for distinguishing cell types. For the trained model, the attention weight matrix $A$ can be used to analyze which genes play important roles in the classification process. Genes are ranked based on weight size, thus identifying those that most contribute to classification decisions. Then, a reverse selection strategy is adopted to remove certain genes that are not significant across all cell types, thereby reducing redundant features and improving the generalizability of the model. Subsequently, the ‘cosg’^1^ package is combined to select the remaining feature genes, based on the characteristic expression patterns of each cell type. Integrating rigorous statistical analysis with advanced computational techniques ensures the selection of features that are not only statistically significant but also computationally robust, thereby capturing the most unique and informative aspects of the data. It is worth noting that the number of differentially expressed genes among cell types is an adjustable variable that can affect the final outcome. Capture efficiency and the number of detected genes are different across spatial transcriptomic platforms. Platforms with deeper sequencing depths and wider gene coverage can provide more accurate results through more differentially expressed genes. Moreover, the filtering of low-quality genes and cells is a key step to influence the mapping quality. Other common parameters are presented in the Supplementary Fig. 9.

**Deep neural network cell typing model**

A lightweight deep neural network model was designed for cell type classification. This fully connected neural network is composed of an input layer, several hidden layers, and an output layer. Given input vector $x_{i}$ (representing gene expression of a single cell), the forward propagation of the model is described as follows, with the structure of the hidden layers as:

$$h_{i}=RELU(W_{i}x_{i}+b_{i})$$

where $W_{i}$ is the weight matrix of the $i$th hidden layer and $b_{i}$ is the corresponding bias vector. $RELU$ (rectified linear unit) is a nonlinear activation function. The structure of the output layer is:
$O=Softmax(W_{o}x_{o}+b_{o}+(W_{r}x_{r}+b_{r}))$

where the Softmax function ensures the output represents a probability distribution, and $W_{r}x_{r}+b_{r}$ is a skip connection learned from ResNet which is directly from the input to output.

To train the model, the cross-entropy loss function is used, defined as:

$$L=-\sum_{i=1}^{N} y_{i}\log\left( o_{i} \right)$$

where $N$ is the number of samples, $y_{i}$ is the one-hot encoded true label, and $o_{i}$ is the prediction probability from the model. In each training epoch, the model performs forward propagation to obtain predictions, followed by loss computation. Subsequently, the gradient with respect to loss is calculated using back propagation, and the model parameters are updated with the Adagrad optimizer. To ensure the generalizability of the model, 80% of the data is used for training, with the remaining 20% reserved for hyperparameter tuning. After every 10 training epochs, model performance on the validation set is evaluated to monitor potential overfitting.

**Establishment of an evaluation system**

A comprehensive evaluation system was established to assess annotation precision. Firstly, we calculated the average gene expression levels for each cell type. This step established a benchmark for the gene expression of specific cell types, enabling detailed comparison between the expression profiles of cells in their actual spatial locations.

$${expr}_{i}=avg(X_{i})$$

where ${expr}_{i}$ represents the average expression of all genes under cell type $i$, $X_{i}$ represents the expression data of all genes in cells of type $i$, and $avg$ represents the averaging operation.

Next, to determine the similarity between the mapping and actual results, four metrics were selected, including Pearson Product-Moment Correlation Coefficient (PPMCC), Cosine Similarity (COS), Structural Similarity Index (SSIM) and Kullback-Leibler Divergence (KL) . Finally, these indicators were combined for overall assessment, using the following formulas:

$${PPMCC}_{j}=\frac{\sum({expr}_{j}-{\mu expr}_{j})(Y_{j}-{\mu y}_{j})}{\sigma_{{expr}_{j}}\sigma_{Y_{j}}}$$

$${Cos(\theta)}_{j}=\frac{{expr}_{j}\cdot Y_{j}}{\left\| {expr}_{j} \right\|\left\| Y_{j} \right\|}$$

$${SSIM}_{j}=\frac{(2{\mu expr}_{j}{\mu y}_{j}+C_{1})(2\sigma_{{expr}_{j}Y_{j}}+C_{2})}{({{\mu expr}_{j}}^{2}+{{\mu y}_{j}}^{2}+C_{1})({\sigma_{{expr}_{j}}}^{2}+{\sigma_{Y_{j}}}^{2}+C_{2})}$$

$${KL}_{j}=\sum{expr}_{j}log\frac{{expr}_{j}}{Y_{j}}$$

In this analytical framework, ${expr}_{j}$ represents the average expression of all genes for the cell type to which cell $j$ belongs, ${\mu expr}_{j}$ represents the average expression level of all genes in the cell type for the cell $j$ being compared, $Y_{j}$ is the specific gene expression amount in the actual spatial transcriptomic data for cell $j$, and ${\mu y}_{j}$ represents the average value of all gene expression amounts in the spatial transcriptomic data for cell $j$. $\sigma_{{expr}_{j}}$，$\sigma_{Y_{j}}$represents their respective standard deviations, and $\left\| {expr}_{j} \right\|$, $\left\| Y_{j} \right\|$ represents their respective magnitudes. These indicators constitute a quantitative assessment of the accuracy of cell-type identification. ${PPMCC}_{j}$, ${Cos(\theta)}_{j}$, ${SSIM}_{j},$ and ${KL}_{j}$ represent the Pearson correlation coefficient, cosine similarity, structural similarity, and degree of dispersion index between the single-cell and spatial transcriptome data for cell $j$.

$${PPMCC}_{final}=avg\left( {PPMCC}_{j} \right)$$

$${Cos\left( \theta\right)}_{final}=avg\left( Cos\left( \theta\right) \right)$$

$${SSIM}_{final}=avg\left( {SSIM}_{j} \right)$$

$${KL}_{final}=avg\left( {KL}_{j} \right)$$

${PPMCC}_{final}$, ${Cos\left( \theta\right)}_{final}$, ${SSIM}_{final}$, and ${KL}_{final}$ represent the average Pearson correlation coefficient, cosine similarity, structural similarity, and degree of dispersion index between the single-cell and spatial transcriptome data. The inclusion of ‘avg’ signifies that the average has been calculated across all cell types, thereby providing a comprehensive assessment of these similarity indicators.

To reduce the impact of the indicators themselves between the indicators and to establish a comprehensive evaluation system, the rankings of the aforementioned indicators were recorded as ${rank}_{cor}$,${rank}_{cos}$, ${rank}_{JS}$, ${rank}_{KL}, \mathrm{and}{rank}_{SSIM}$^2^.

The accuracy rank score is calculated as:

$${Rank Score=\frac{1}{4}(rank}_{cor}+{rank}_{cos}+{rank}_{KL}+{rank}_{SSIM})$$

This evaluation method ensures that each indicator equally influences the final evaluation result, without any single indicator disproportionately affecting the overall assessment due to significant numerical fluctuations. Next, for the comparison between different algorithms, we will normalize the Rank Score and get Acc Score:

$${Acc Score}_{i}=\frac{{Rank Score}_{i}-min(Rank Score)}{max(Rank Score)-min(Rank Score)}$$

For a dataset, the method with the highest $Acc Score$ value has the best performance among the methods.

**Weight calculation of cell types**

In this study, a method to calculate cell-type weights was adopted. Firstly, for each cell type, the previously mentioned feature selection method was used to identify the corresponding feature genes. The quantity of expressed feature genes serves as an indicator of the spatial distribution of each cell type. When a cell expresses a specific feature gene, its weight increases by 1. By performing this operation on the feature genes of all cell types, we can obtain the distribution weights of each cell type. The formula is as follows:
$W_{i}=\sum_{j=1}^{n} x_{j}$

Where $W_{i}$ represents the weight of the $i$th cell type, $n$ is the number of feature genes corresponding to the cell type, and $x_{j}$ is an indicator function, taking the value of 1 when the *i*th cell expresses the $j$th feature gene and 0 otherwise. In this way, we can quantify the importance or occurrence rate of each cell type and make more targeted comparisons and evaluations of cell types identified by DSCT based on their weights.

**Confusion matrix heatmap**

To evaluate the performance of cell-type predictions in our spatial transcriptomic data, confusion matrix analysis was used (Supplementary Fig. 5). A confusion matrix is a specific table format designed to visualize and assess the performance of a method by showing how well the model predicts categories and which categories are more likely to be misclassified. Firstly, we used known cerebral cortex (CTX) cell-type labels (human and mouse) from the MERFISH platform as the previous annotated data, then predicted new CTX cell-type labels (human and mouse) using DSCT. A confusion matrix was constructed based on the previous annotated data and predicted cell-type labels. Each matrix element represented a cell count between a specific true category and a predicted category. A heatmap was used to visualize the confusion matrix, where rows represented true cell types and columns indicated predicted cell types. Color intensity represented the correspondence level, with deeper colors denoting a higher degree of matching and paler colors denoting a lower degree or absence of matching.

**Parameters of tested mapping algorithms**

SpatialDWLS: The SpatialDWLS code was obtained from https://github.com/rdong08/spatialDWLS_dataset. The parameters were set to method = ‘scran’, expression_values = ‘normalized’, and cluster_column = ‘leiden_clus’ for the ‘findMarkers_one_vs_all’ function, and resolution = 0.4 and n_iterations = 100 for the ‘doLeidenCluster’ function.

RCTD: The RCTD code was obtained from https://github.com/dmcable/spacexr, and integrated into spacexr (v2.0.0). The parameters were set to doublet_mode = ‘full’ and ‘n_max_cells’ = 10 000 for processing reference data.

Seurat: Following the instructions on the Seurat 3.2 website: https://satijalab.org/seurat/archive/v3.2/integration.html, the parameters were set to dim = 1:20, normalization.method = ‘SCT’, and reference.reduction = ‘pca’ for the ‘FindTransferAnchors’ function.

Tangram: The Tangram code was obtained from https://github.com/broadinstitute/Tangram. The parameters were set to mode = ‘cells’, density_prior = ‘rna_count_based’, and num_epochs = 100 for the ‘map_cells_to_space’ function.

Cell2location: The Cell2location code was obtained from https://github.com/BayraktarLab/cell2location. The parameters were set to max_epochs = 300, batch_size = 3000, and train_size = 1, lr = 0.002 for training.

DestVI: The DestVI code was obtained from https://github.com/scverse/scvi-tools. The parameters were set to max_epochs = 300 for training the snRNA-seq model, while the spatial model was trained for 2 000 epochs, with a learning rate of 0.001.

Spatial-ID: Following instructions from the website https://github.com/STOmics/SpatialID/tree/main/spatialid, the parameters for mapping labels from the reference to spatial data were set to pca_dim = 200, k_graph = 30, edge_weight = True, epochs = 200, w_cls = 20, w_dae = 1, and w_gae = 1.

**Computational resources**

All computational analyses presented in the paper were conducted on a system equipped with 128 GB of RAM, an Intel Core i7-12700KF processor with 12 cores operating at 3.6 GHz, and an NVIDIA GeForce RTX 3080 with 12 GB of video memory. The operating system used was Windows 11. To support the Spatial-ID application, the following python (v3.9) packages were required: cosg==1.0.1, numpy==1.23.4, numba==0.56.4, pandas==1.5.2, scipy==1.9.3, matplotlib==3.6.2, seaborn==0.12.1, scikit-lean==1.2.0, torch==1.13.1+cu116, torchgeometric==0.11.0, torchvision==0.14.1+cu116, scanpy==1.8.1.

1 Dai, M., Pei, X. & Wang, X. J. Accurate and fast cell marker gene identification with COSG. *Brief Bioinform* **23** (2022). <https://doi.org:10.1093/bib/bbab579>

2 Li, B. *et al.* Benchmarking spatial and single-cell transcriptomics integration methods for transcript distribution prediction and cell type deconvolution. *Nat Methods* **19**, 662-670 (2022). <https://doi.org:10.1038/s41592-022-01480-9>
