# Supplementary material for: DSCT: a novel deep-learning framework for rapid and accurate spatial transcriptomic cell typing
Source: Natl Sci Rev. 2025 Jan 28;12(5):nwaf030. doi: 10.1093/nsr/nwaf030 (PMC12045154; doi:10.1093/nsr/nwaf030)
Supplement: nwaf030_Supplemental_Files [file nwaf030_supplemental_files.zip › Supplementary Table 1.pdf]

Supplementary Table 1: CPU and GPU runtimes of different cell-type prediction methods

Methods / Datasets	DSCT		DestVI		SpatialID		Cell2location	
	CPU Time/s	GPU Time/s	CPU Time/s	GPU Time/s	CPU Time/s	GPU Time/s	CPU Time/s	GPU Time/s
CB_Stereo-seq	3.8	2.2	673.0	613.0	1523.0	330.0	2640.0	706.0
OB_Stereo-seq	29.5	15.5	13428.0	4323.0	11349.0	2080.0	14532.0	4516.0
HIP_Stereo-seq	14.8	13.1	3078.0	762.0	927.0	203.0	2251.0	672.0
HIP_Slide_seq	19.8	14.2	2806.0	956.0	1301.0	238.0	2872.0	935.0
HIP_10x	14.9	12.7	2005.0	578.0	869.0	187.0	1991.0	679.0
HIP_STARmap	9.1	8.2	1211.0	546.0	975.0	199.0	2367.0	576.0
CTX_MERFISH_human	17.4	13.6	1547.0	470.0	411.0	93.0	1457.0	468.0
CTX_MERFISH_mouse	3.1	2.6	1201.0	460.0	360.0	87.0	1007.0	308.0
cancer	11.3	3.23s	12804.3	4450.6	2542	824.5s	15661.0	6106.0
HPF 36 (cluster)	281.2	27.1	18324.7	2317.9	828453.0	1204.2	139324.0	3670.0
HPF 37 (cluster)	290.4	28.5	17467.8	2179.7	817241.0	1102.8	142923.0	3778.0
HPF 36 (supertype)	89.6	19.9	18778.8	2214.0	838432.0	1067.3	111242.0	3111.0
HPF 37 (supertype)	82.6	16.3	17934.6	2117.2	817628.0	1115.7	114121.0	2907.0
HPF 36 (subclass)	66.1	13.5	18154.3	2298.1	815424.0	1086.2	103325.0	2947.0
HPF 37 (subclass)	64.3	14.3	18273.3	2171.9	808112.0	1090.6	105844.0	2808.0
HPF 36 (class)	69.7	12.9	18724.8	2181.6	815732.0	1087.8	104043.0	3052.0
HPF 37 (class)	64.1	13.5	18355.6	2100.7	864457.0	1045.0	105482.0	2817.0

(Continued)

Methods / Datasets	Tangram		RCTD		Seurat		SpatialDWLS	
	CPU Time/s	GPU Time/s	CPU Time/s	no GPU	CPU Time/s	no GPU	CPU Time/s	no GPU
CB_Stereo-seq	431.0	15.6	846.1		120.4		2520.9	
OB_Stereo-seq	8761.0	Nan	762.0		762.3		8580.5	
HIP_Stereo-seq	1074.0	13.9	499.5		420.7		2160.3	
HIP_Slide_seq	11425.0	Nan	1512.2		536.6		3720.5	
HIP_10x	259.0	14.3	298.0		396.0		2040.7	
HIP_STARmap	2357.0	Nan	465.4		445.1		2820.3	
CTX_MERFISH_human	237.0	15.7	99.6		193.2		960.2	
CTX_MERFISH_mouse	406.0	18.8	131.9		118.4		901.4	
cancer	3242.5	Nan	11521.2		324.6		Nan	
HPF 36 (cluster)	3013.5	Nan	68040.3		2400.6		Nan	
HPF 37 (cluster)	2727.5	Nan	53281.7		2581.6		Nan	
HPF 36 (supertype)	1816.0	Nan	9722.1		2676.8		Nan	
HPF 37 (supertype)	1431.7	Nan	7922.3		2706.1		Nan	
HPF 36 (subclass)	1344.5	Nan	1458.3		2508.2		Nan	
HPF 37 (subclass)	1192.2	Nan	1206.4		2406.3		Nan	
HPF 36 (class)	1423.6	Nan	330.9		3000.7		Nan	
HPF 37 (class)	1045.1	Nan	276.8		2682.5		Nan	
