# Supplementary material for: DSCT: a novel deep-learning framework for rapid and accurate spatial transcriptomic cell typing
Source: Natl Sci Rev. 2025 Jan 28;12(5):nwaf030. doi: 10.1093/nsr/nwaf030 (PMC12045154; doi:10.1093/nsr/nwaf030)
Supplement: nwaf030_Supplemental_Files [file nwaf030_supplemental_files.zip › Supplementary Table 2.pdf]

Supplementary Table2: Random memory and video random memory costs of different cell-type prediction methods

Datasets \ Methods	DSCT		DestVI		SpatialID		Cell2location	
	RAM/GB	VRAM/GB	RAM/GB	VRAM/GB	RAM/GB	VRAM/GB	RAM/GB	VRAM/GB
CB_Stereo-seq	2.6	1.5	3.1	1	2.9	1.4	3.6	3.9
OB_Stereo-seq	8.2	1.9	8.1	1.2	6.2	2.2	6.1	3.8
HIP_Stereo-seq	1.5	1.5	11.7	1.4	14.4	2.2	3.9	4.7
HIP_Slide_seq	8.1	2.4	7.1	0.9	10.8	2.7	3.7	3.4
HIP_10x	7.2	1.8	6.9	1.2	7.7	2.6	4.2	3.3
HIP_STARmap	5.1	1.3	3.3	0.9	7.9	2.7	3.9	3.4
CTX_MERFISH_human	11.5	1	5.2	1	17.8	3	3.5	4.1
CTX_MERFISH_mouse	2.6	1	4.8	0.9	19.2	2.3	3.2	3.3
cancer	2.4	1.4	3.4	0.7	8.2	6.6	5.7	2.8
HPF 36 (cluster)	26.3	2.5	14.9	0.5	87.5	1.5	50.4	5.8
HPF 37 (cluster)	25.1	2.6	14.8	0.5	87.6	1.5	49.4	5.8
HPF 36 (supertype)	24.6	1.4	14.9	0.5	87.5	1.5	51.3	5.1
HPF 37 (supertype)	23.8	1.4	14.8	0.5	87.6	1.5	49.7	5.1
HPF 36 (subclass)	29.6	1.2	14.9	0.5	87.5	1.5	49.7	4.7
HPF 37 (subclass)	28.7	1.2	14.8	0.5	87.6	1.5	51.9	4.7
HPF 36 (class)	26.6	1.1	14.9	0.5	87.5	1.5	49.4	4.7
HPF 37 (class)	24.6	1.1	14.8	0.5	87.6	1.5	48.7	4.7

(Continued)

Datasets \ Methods	Tangram		RCTD		Seurat		SpatialDWLS	
	RAM/GB	VRAM/GB	RAM/GB	no VRAM	RAM/GB	no VRAM	RAM/GB	no VRAM
CB_Stereo-seq	9.1	1.5	9.5		6.4		13.9	
OB_Stereo-seq	61.5	31	26.7		58.3		123.4	
HIP_Stereo-seq	13.2	6.6	25.7		43.8		99.4	
HIP_Slide_seq	34.9	27	25.8		54.7		118.9	
HIP_10x	6.6	0.9	22.4		46.5		105.1	
HIP_STARmap	14.3	27.7	19		48.7		83.9	
CTX_MERFISH_human	5.7	1.2	13.4		44.3		40.5	
CTX_MERFISH_mouse	3.4	2.7	15.2		38.7		37.4	
cancer	156.7	Nan	41.7		17.4		Nan	
HPF 36 (cluster)	48.3	Nan	100.4		153.5		Nan	
HPF 37 (cluster)	51.3	Nan	99.8		153.5		Nan	
HPF 36 (supertype)	50.3	Nan	92.2		153.6		Nan	
HPF 37 (supertype)	49.4	Nan	93.6		152.8		Nan	
HPF 36 (subclass)	49.3	Nan	77.3		152.4		Nan	
HPF 37 (subclass)	48.4	Nan	78.4		153.7		Nan	
HPF 36 (class)	49.6	Nan	75.5		155.8		Nan	
HPF 37 (class)	47.4	Nan	74.2		154.6		Nan	
